# Synergy between reactive manganese species and enzymes drives litter decomposition hotspots

**DOI:** 10.1038/s41467-026-75920-x

**Published:** 2026-07-25

**Authors:** Nathan A. Chin, Luiz A. Domeignoz-Horta, Xiao Jun A. Liu, Morris E. Jones, Ravi Ranjan, Malak Tfaily, Kristen M. DeAngelis, Marco Keiluweit

**Affiliations:** 1 Institute of Earth Surface Dynamics, Faculty of Geosciences and Environment, University of Lausanne, Lausanne, Switzerland; 2 French National Institute for Agriculture, Food, and Environment, Paris, France; 3 Department of Biological Sciences, Louisiana State University, Baton Rouge, LA USA; 4 College of Health and Natural Sciences, Franklin Pierce University, Rindge, NH USA; 5 Genomics Resource Laboratory, Institute for Applied Life Sciences, University of Massachusetts, Amherst, MA USA; 6 Department of Environmental Science, University of Arizona, Tucson, AZ USA; 7 Microbiology Department, University of Massachusetts, Amherst, MA USA; 8 Present Address: Yale Center for Genome Analysis, Department of Genetics, Yale University, New Haven, CT USA

**Keywords:** Carbon cycle, Ecosystem ecology, Geochemistry

## Abstract

Oxidative decomposition of plant litter is a central control on carbon storage and productivity in terrestrial ecosystems. Although this process is commonly assumed to be greatest under oxic conditions, recent findings highlight redox-sensitive metals as potent, but poorly understood, mediators. Using controlled soil reactors, we show that stimulating microbial production of reactive manganese(III) at oxic–anoxic interfaces simultaneously increased the expression of diverse litter-decomposing enzymes; together, these metal and enzyme catalysts acted synergistically to accelerate litter decomposition. This 29% increase in decomposition was best predicted by multi-fold increases in the expression of putative Mn(III)-forming and litter-degrading enzymes at the interface, rather than by patterns in strictly oxic or anoxic zones. These results provide evidence to the growing body of research challenging the assumption that oxidative decomposition is maximized under fully oxic conditions. Instead, they reveal a tight interplay among oxygen supply, reactive metal regeneration, and enzyme activity that renders oxic–anoxic interfaces hotspots of litter decomposition in soils.

## Abstract (web summary)

Oxic–anoxic interfaces promote microbial production of reactive manganese species that enhance expression of diverse litter-degrading enzymes and accelerate decomposition, yielding rates far exceeding those in fully oxic or anoxic environments.

## Introduction

Oxidative decomposition of plant litter in soils is a key ecosystem process regulating nutrient cycling, carbon storage, and greenhouse gas emissions^1,2^. A critical rate-limiting step is the breakdown of intact lignocellulose within litter, with lignin content often predicting decomposition rates^3,4^. The depolymerization of lignin has traditionally been attributed to highly specialized oxidative enzymes^5,6^ that require molecular oxygen, leading to the assumption that oxic conditions universally favor more rapid lignin decomposition due to greater oxidative enzyme activity^7^. Yet, soils frequently experience dynamic transitions between oxic and anoxic states, generating decomposition hot spots and hot moments that have been linked to the cycling and regeneration of redox-active metal catalysts, such as iron or manganese (Mn)^8,9^. The mechanism underlying the interplay between enzymatic activity and redox-active metals—acting as active centers, co-factors and reactive intermediates in the Earth system—remains a major scientific challenge^10^, particularly in the context of litter decomposition^11,12^. Without a mechanistic understanding of these synergies, predictive models will struggle to capture how litter decomposition responds to alterations of soil redox conditions, as expected with climate change^13^.

Mn is an abundant and redox-active metal that consistently predicts litter decomposition rates across diverse ecosystems, including coniferous^14,15^ and deciduous forests^16,17^, peatlands^18^, aquatic systems^19^, and agroecosystems^20^, thereby influencing long-term soil carbon storage^21,22^. This effect is often attributed to Mn(III)–ligand complexes, among the most potent oxidants in the environment^23^. In soils, lignolytic fungi are thought to produce these transient yet highly reactive Mn(III) species via enzymatic oxidation of dissolved Mn(II)^24,25^, though support has also been shown for bacterially-mediated oxidation to potentially play a role in soils as well^26^. When stabilized by organic chelators, Mn(III) not only oxidizes a broad range of organic and inorganic substrates^27,28^, but is also considered particularly effective at lignin depolymerization^29^. Its small molecular size is thought to allow it to penetrate intact lignocellulosic structures, conferring an advantage over larger extracellular oxidative enzymes^24^. Using spectroscopic and microscopic tools, we recently demonstrated a link between Mn(II) oxidation and long-term litter decomposition in soils^30^. Yet, attempts to directly test the positive effect of Mn availability on decomposition have yielded ambiguous or even contradictory results^31,32^, despite growing evidence that Mn(II) availability is coupled to Mn oxidation and lignin decomposition^33,34^, likely because experimental conditions did not sufficiently favor, or even suppressed, microbial Mn(III) formation^35^. A mechanistic understanding of the environmental controls on and microbial drivers of Mn(III) formation is therefore essential to resolve the Mn-dependence of litter decomposition at ecosystem scales.

Until recently, the presence of transient and reactive Mn(III) species in the environment was only speculative, but oxic-anoxic interfaces in both terrestrial and aquatic ecosystems have emerged as hotspots for their formation. Using a spectrophotometric approach, Luther and co-authors^36^ detected the greatest Mn(III) concentrations at the oxic-anoxic interfaces in aquatic^37^ and sedimentary environments^38^, a general pattern confirmed in soil ecosystems^39^. Using soil reactors, which establish and maintain stable oxygen gradients to identify biogeochemical hotspots, an experimental study demonstrated extensive biological Mn(II) oxidation at oxic-anoxic interfaces^40^. Although Mn(III) was not measured, soil-borne Mn(II)-oxidizing fungi preferentially colonized the interface. These fungi seemingly harnessed Mn(II) generated through Mn(III,IV) oxide reduction in the anoxic zone while maintaining access to O_2_ from the oxic zone^34^. Similar soil reactor experiments combined with X-ray absorption spectroscopy previously demonstrated that Mn(III) indeed forms at oxic-anoxic interfaces^41^, but the underlying microbial drivers of Mn(III) formation and its impact on decomposition remained inconclusive. Oxic-anoxic interfaces are prevalent in soil environmnents^40,42,43^, including aboveground litter^44^, and are increasingly regarded as decomposition hotspots^45,46^. However, it remains unclear to what extent litter decomposition across these interfaces might respond to accelerated Mn redox cycling, including the formation of reactive Mn(III) species.

While Mn(III) in soils can also arise from Mn(IV) reduction^47^ or abiotic Mn(II) oxidation^48^, mounting evidence points to microbial Mn(II) oxidation as the dominant pathway^49^. Extensive work with bacterial and fungal isolates has identified two groups of oxygen-dependent enzymes as direct catalysts of Mn(II) oxidation—class II peroxidases (e.g., manganese peroxidase, or MnP) and laccases^24,50^—alongside indirect pathways mediated by ubiquitous enzymatically generated reactive oxygen species^51,52^. However, previous efforts to resolve the interactive effect between enzymes and metal catalysts in soils have been hindered by the non-specific nature of bioassays used to assess oxidative enzyme activities^8,53^, which, at best, can only provide an estimate of the cumulative potential oxidative effect of these intricate reactions. Recent proteomics studies have demonstrated that Mn(II)-oxidizing activity in fungal isolates correlates with the presence of specific enzymes^54,55^, raising the possibility that in situ Mn(III) formation rates in soil systems can be quantitatively linked to the expression of select enzymes using advanced molecular techniques. To date, however, the mechanistic link between enzymatic Mn(II) oxidation and the formation of the potent oxidant Mn(III), as well as its role in litter decomposition, remains unresolved.

**Fig. 1:**
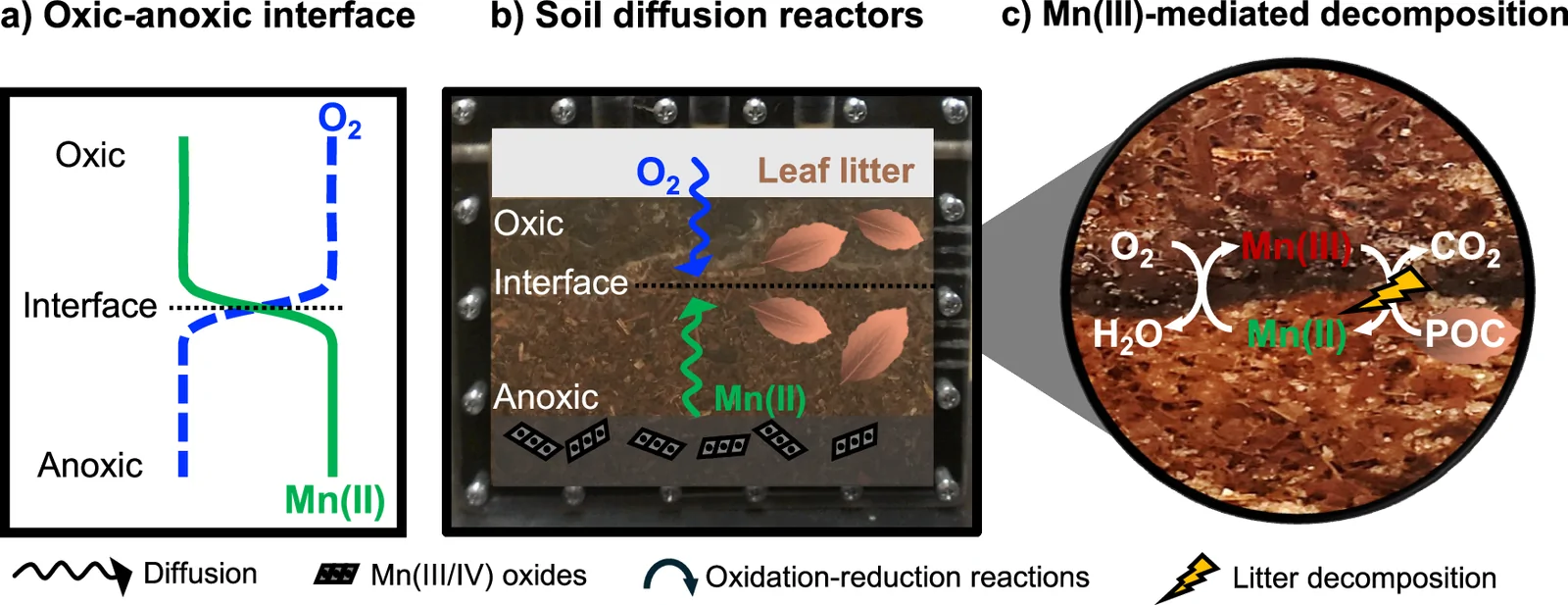
Resolving manganese(III)-mediated litter decomposition at oxic-anoxic interfaces within diffusion reactors. **a** Ubiquitous oxic-anoxic gradients in soils result in opposing diffusive gradients of Mn(II) and O_2_, with both overlapping only at the interface. **b** We recreated these oxic-anoxic interfaces within soil diffusion reactors and manipulated Mn(II) supply towards the interface via the reductive dissolution of Mn(III/IV) oxides added to the bottom layer (grey zone). During the experiment, Mn(III/IV) oxides in the anoxic layer were reductively reduced, allowing for diffusion and provision of Mn(II) to the interface. **c** The subsequent oxidation of Mn(II) to reactive Mn(III) species was hypothesized to facilitate the decomposition of fresh litter at these oxic-anoxic interfaces. We expected acceleration of litter decomposition with increased Mn(III) formation at the interface. Particulate organic carbon (POC) denotes insoluble structural components of the litter that needs to be depolymerized prior to facilitate microbial conversion to CO_2_. The brown leaf icon used in (**b**) is reprinted from Creazilla, “autumn leaf brown fall color,” public domain (CC0).

Here, we aim to resolve the role of Mn availability in regulating microbially mediated Mn(III) formation across oxic–anoxic interfaces and its consequences for litter decomposition. We hypothesize that enhanced Mn cycling across oxic-anoxic interfaces would stimulate enzymatic Mn(III) formation, creating “hotspots” of litter decomposition. To test this, we establish soil diffusion reactors^41^ (Fig. 1) that generate stable, opposing diffusive gradients of O_2_ and Mn(II) gradients across well-defined oxic-anoxic interfaces (Fig. 1a), while allowing precise manipulation of Mn(II) supply. We vary Mn(II) supply towards the interface by adding different amounts of Mn oxide-coated sand (birnessite, a mixed-valence Mn(III/IV) oxide^56^) to the anoxic zone at the bottom of the reactors (Fig. 1b). Reductive dissolution of Mn(III/IV) oxides in the anoxic zone are intended to create a diffusive gradient of Mn(II) towards the interface. Reactors containing forest broadleaf litter mixed with sand are incubated over a 90-day period with no, low, or high Mn oxide additions. Destructive sampling after 7, 30, and 90 days allows us to spatiotemporally resolve Mn(III) dynamics and associated changes in litter decomposition and microbial activity across the oxic–anoxic gradients (Fig. 1c) using a combination of spectroscopic, molecular biology, and mass spectrometry approaches. The resulting findings highlight the complex interplay between oxygen supply, regeneration of reactive Mn(III) species, and enzyme activities that govern the emergence of “hotspots” of litter decomposition along oxic-anoxic interfaces in soils.

## Results

### Transient hotspots of Mn(III) formation across oxic-anoxic interfaces

**Fig. 2:**
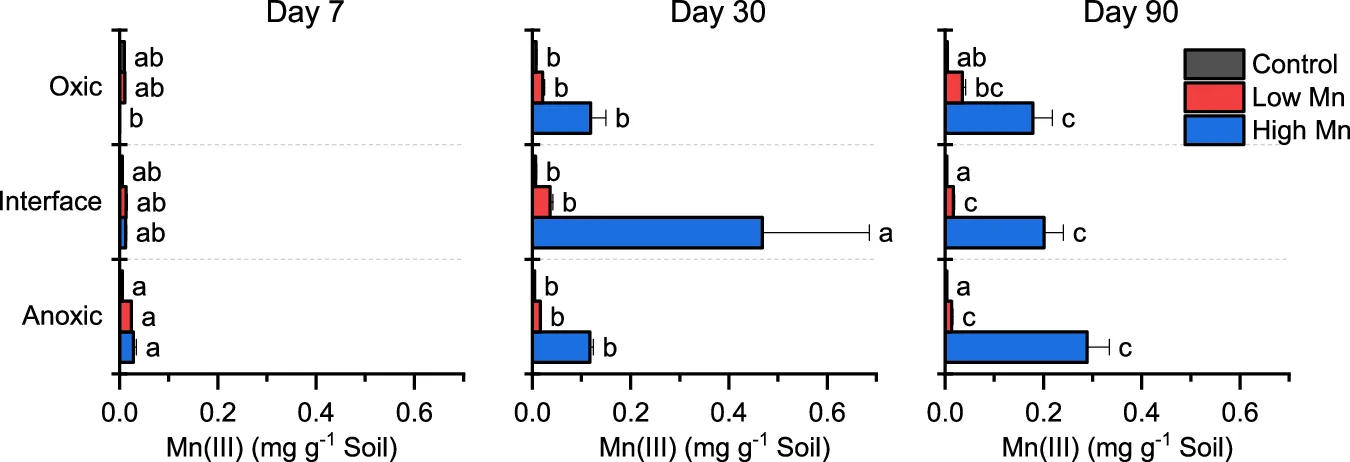
Manganese(III) formation across the oxic-anoxic interface as a function of Mn supply. Absolute Mn(III) concentrations were calculated by multiplying the fraction of Mn(III) as determined by XANES with the total extractable Mn as determined by sequential extractions (Supplementary Table 6). Error bars denote the standard error of the mean (*n* = 4), while differing letters within each timepoint represent statistical differences between treatment and location at the *p* < 0.05 level (2-way ANOVA, Tukey test).

Across all Mn treatments, X-ray absorption near edge structure (XANES) spectroscopy in combination with wet-chemical extractions showed that the greatest Mn(III) concentrations occurred at the oxic-anoxic interface on day 30 of the experiment (Fig. 2). This spatial peak in Mn(III) concentrations was significant for the high Mn treatment (*p* < 0.001), and resulted in a visually distinct dark band of oxidized Mn at the oxic-anoxic interface (Fig. 1c). This peak in Mn(III) concentrations after 30 days was consistent with a maxima in pyrophosphate extractable Mn (Supplementary Fig. 3) and dissolved Mn in the pore water (Supplementary Fig. 4), which generally is well-correlated to the presence of Mn(III)^30,53^. Conversely, we found no significant differences in total Mn(III) and pyrophosphate extractable Mn across the oxic-anoxic interface at day 7 and day 90 in the high Mn treatment. Combined, these results show that the oxic-anoxic interface developed into a transient hotspot of Mn(III) formation after 30 days, particularly under high Mn availability.

### Enhanced Mn(III) formation increased aromatic compound decomposition and CO_2_ production

**Fig. 3:**
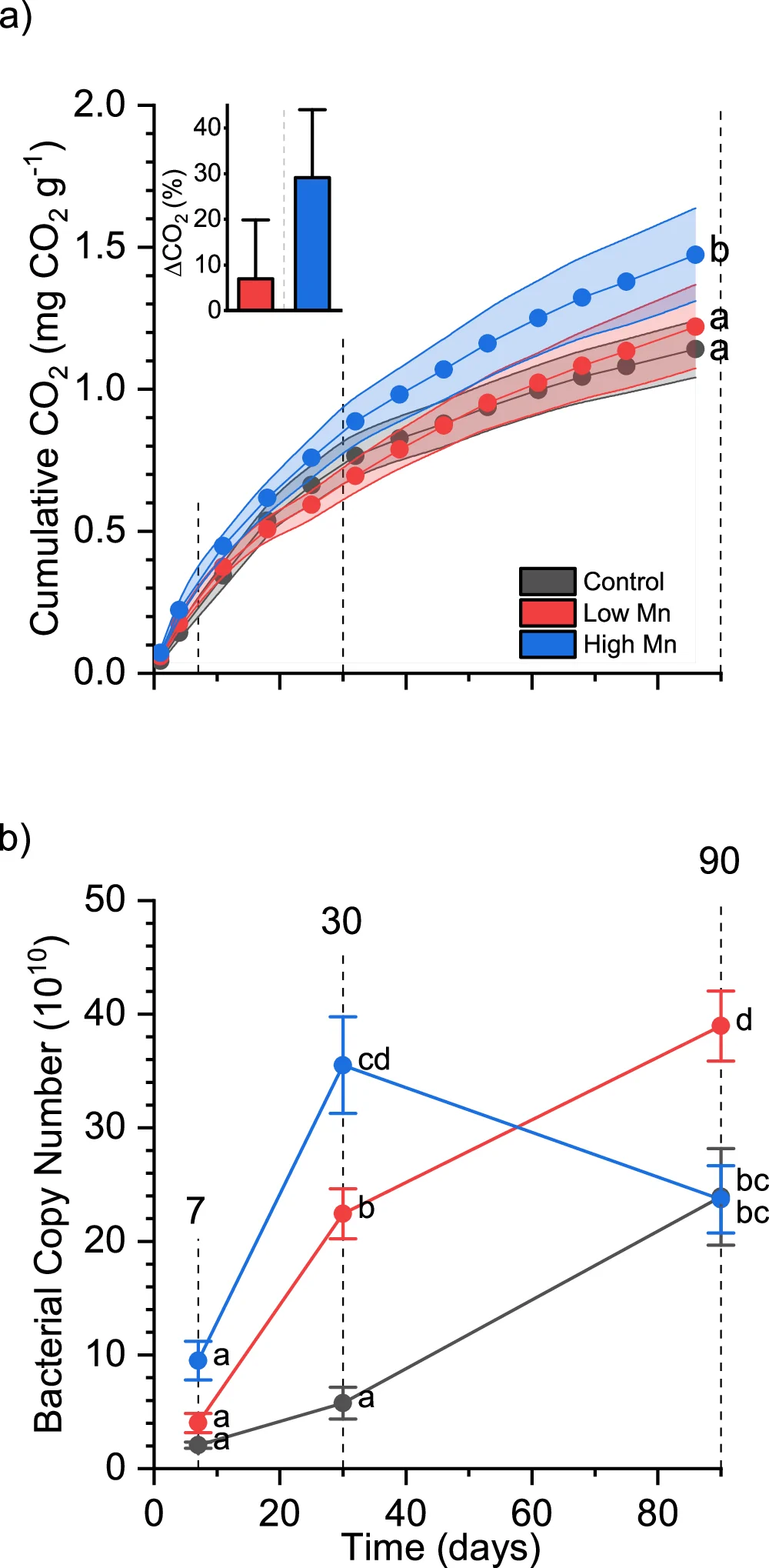
Effect of manganese availability on litter decomposition and microbial growth. **a** Cumulative CO_2_ production as a measure of microbial decomposition of litter over the course of the experiment. Shaded regions represent the calculated propagated standard error of the mean (*n* = 4). The insert represents the difference (%) in total cumulative CO_2_ production between low and high Mn treatments and the control treatment at the end of the experiment. Dotted lines denote days at which reactors were harvested for subsequent analyses. Letters denote statistical differences between treatments at *p* < 0.05 on models used to fit the respiration data (F-test). **b** Changes in bacterial abundance averaged across all sampling zones for each treatment were determined using qPCR. Error bars denote the standard error of the mean (*n* = 4), while letters denote statistical differences between treatment and timepoint at the *p* < 0.05 level (2-way ANOVA, Tukey test). Full data for qPCR results can be found in Supplementary Fig. 5. Dashed lines denote destructive harvest points.

Enhanced Mn(III) formation in Mn-amended reactors coincided with significantly greater CO_2_ production (Fig. 3a). After around 30 days, CO_2_ production rates in the high Mn treatment accelerated, resulting in a 29% increase in cumulative CO_2_ production at the end of the experiment compared to the control (Fig. 3a, inset). This acceleration in CO_2_ production at day 30 coincided with shifts in the microbial community, specifically a significant increase in bacterial abundance as measured by qPCR within the low Mn (approx. +450%, *p* < 0.001, Fig. 3b and Supplementary Fig. 5a) and high Mn treatments (+270%, *p* < 0.001), both of which were significantly enhanced by Mn addition relative to the control (+290% and +520%, respectively, *p* < 0.001). In contrast, fungal abundance showed a significant decline at day 30 for the control and high Mn treatment (−620% and −710%, respectively, *p* < 0.001, Supplementary Fig. 5b). Because Mn(III) formation (Fig. 2), CO_2_ production rates (Fig. 3a), and microbial community shifts (Fig. 3b) in our high Mn treatment peaked at day 30, we focused our detailed molecular analyses on this time point.

High-resolution mass spectrometry analysis of extracted organic matter showed that enhanced Mn availability significantly diminished abundances of aromatic or ring-like compound classes. Both low and high Mn additions significantly decreased the relative abundance of compounds classified as condensed hydrocarbon-like (−26 to −64%, *p* < 0.05, Supplementary Fig. 1a) and tannin-like (−50 to −69%, *p* < 0.05, Supplementary Fig. 1b) compounds compared to the control. At day 30, the abundances of condensed hydrocarbon-like (Supplementary Fig. 2a; *p* < 0.001) and tannin-like (Supplementary Fig. 2b; *p* < 0.0001) compounds were significantly and negatively correlated with Mn(III) concentrations. Because tannins and condensed hydrocarbons contain extensive aromatic moieties, these trends indicate that enhanced Mn(III) formation promoted the oxidative depolymerization of aromatic compounds within litter.

### Elevated Mn availability enhanced gene expression at the oxic-anoxic interface

**Fig. 4:**
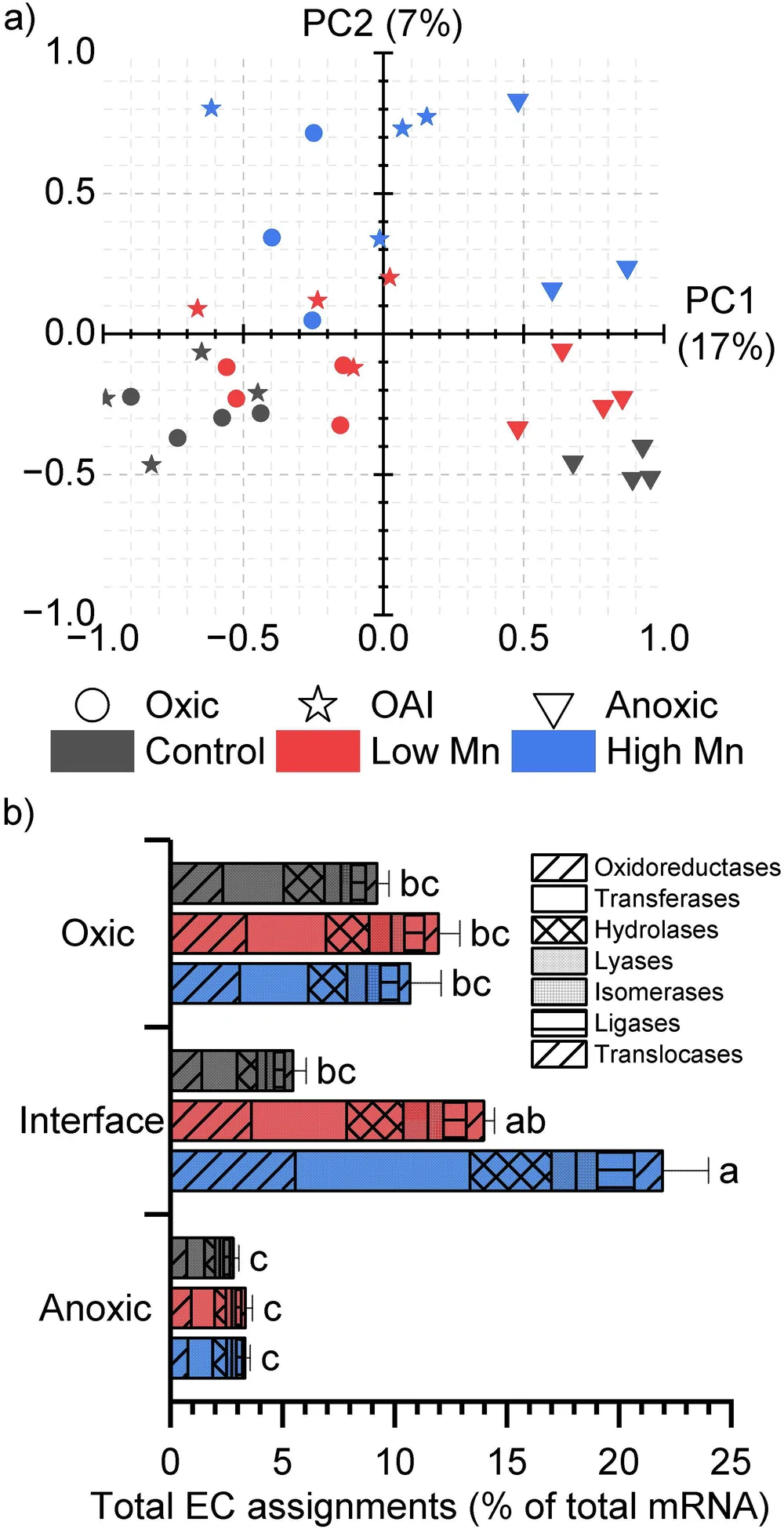
Manganese-dependent gene expression across the oxic-anoxic interface (day 30). **a** Principal component analysis of enzymatic gene expression libraries within sample reactors showing a distinct clustering by Mn treatments and sampling zone. The distance between sampling points represents their (dis)similarities, where samples closer together are similar, while samples further apart or not similar. ANOSIM analysis conducted on the gene expression libraries to determine statistical significance (*p* < 0.05) between different groups are found in Supplementary Table 1. **b** Differential expression of functional enzyme classes based on treatment and location along the oxygen gradient. The relative expression of identified genes were categorized between oxidoreductases, transferases, hydrolases, lyases, isomerases, ligases, and translocases. Error bars denote the propagated standard error of the mean (*n* = 4), while letters denote statistical differences between Mn treatment and location (2-way ANOVA, Tukey test).

Enhanced Mn availability increased the expression of genes transcribed from a broad suite of enzyme categories as determined by metatranscriptomics, particularly at the oxic-anoxic interface (Fig. 4a). Principal component analysis showed that gene expression profiles at day 30 clearly separated across the oxic-anoxic transition (PC1) and Mn treatments (PC2). While gene expression profiles in the anoxic zone remained the most similar, expression profiles in the oxic zone and at the interface showed the largest separation. An ANOSIM of gene expression profiles showed the greatest dissimilarity for the interface under high Mn availability compared to control treatments (*p* = 0.029, Supplementary Table 1).

Total expression of assigned genes was consistently lowest in the anoxic zone, and expression gradually increased from anoxic to the oxic zones in the control treatment, as expected with greater aerobic microbial activity (Fig. 4b). In contrast, however, enhanced Mn availability caused a dramatic increase in gene expression at the interface. Not only did total gene expression at the interface in the high Mn treatment exceed that of the control treatment by a factor of four, but it was also twice as high as in the oxic zone. This significant increase in total gene expression at the interface in response to enhanced Mn availability was primarily driven by increased expression of oxidoreductases (+300%, *p* < 0.001), transferases (+395%, *p* < 0.001), and hydrolases (+300%, *p* = 0.008) (Fig. 4b and Supplementary Table 2c).

### Linking Mn(III) formation, gene expression, and decomposition

**Fig. 5:**
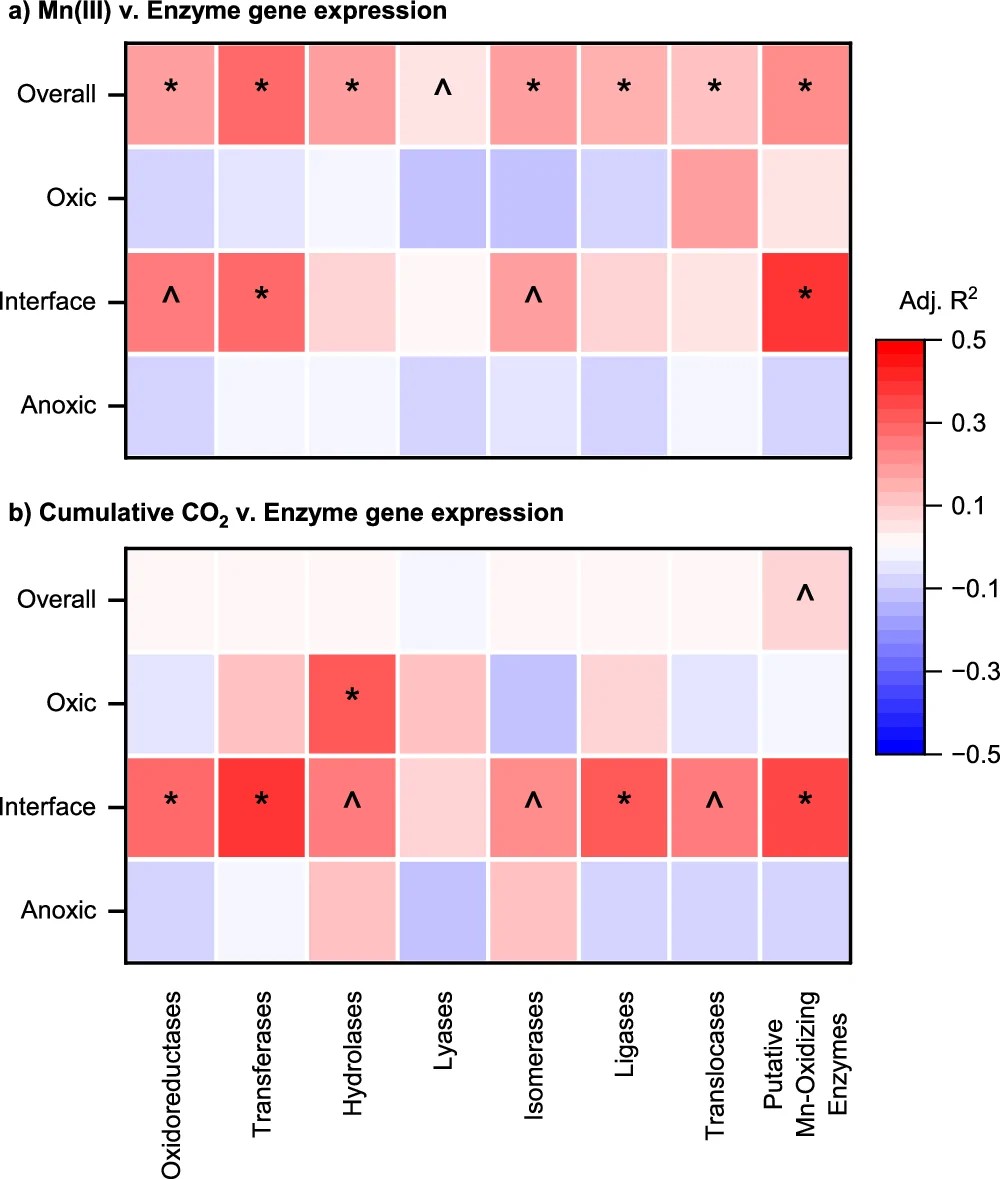
Covariation of reactive manganese(III) species and CO_2_ production with key enzyme classes across the oxic-anoxic interface. Correlation matrix between the relative expression of principal enzyme categories (ECs) to **a** measured Mn(III) concentrations and **b** cumulative CO_2_ production at the end of the incubation period. Correlations for both matrices were conducted across all zones sampled from the reactor (“overall”), and then for each zone (“oxic,” “interface,” and “anoxic”) individually. Coloration corresponds to the adjusted *R*^2^, while * denotes statistically significant correlations (*p* < 0.05) and ^ denotes marginally significant correlations (*p* < 0.10) determined using the F-statistic on the fitted regression line.

Linear regressions between expressed genes and Mn(III) concentrations identified potential enzymatic drivers of Mn(III) formation (Fig. 5a). Mn(III) concentration was most strongly correlated to oxidoreductases (*R*^2^ = 0.198, *p* = 0.004) and transferases (*R*^2^ = 0.287, *p* < 0.001). We found no significant correlations between Mn(III) concentrations and single enzymes with known capacity to directly oxidize Mn(II) (e.g., Mn peroxidase). In contrast, however, Mn(III) concentrations were strongly correlated to the expression of enzymes with putative roles in direct or indirect Mn(II) oxidation (*R*^2^ = 0.205, *p* = 0.004), for example, via ROS or other intermediates (see Supplementary Table 4 for a curated list) (Fig. 5a). Regression analyses for each zone individually revealed that this strong relationship between Mn(III) concentration and the expression of oxidoreductases, transferases and putatively Mn-oxidizing enzymes was exclusive to the interface, and not found within either the oxic or anoxic zones (Fig. 5a).

Finally, to examine if the upregulation of specific genes is related to enhanced litter decomposition, we conducted linear regressions between enzymatic gene expression patterns to cumulative CO_2_ production at the conclusion of the experiment (Fig. 5b). Notably, only the expression of oxidoreductases, transferases, ligases and putatively Mn-oxidizing enzymes at the interface was significantly and positively correlated to CO_2_ production. Apart from hydrolases in the oxic zone, gene expression within the oxic and anoxic zones was not significantly correlated to CO_2_ production (Fig. 5b). In sum, these results directly link Mn(III) formation and the activity of putative Mn-oxidizing and litter-degrading enzymes at the oxic-anoxic interface with overall CO_2_ production.

## Discussion

Our results demonstrate a mechanistic linkage between Mn(III) formation, potential enzyme activity, and litter decomposition at oxic–anoxic interfaces. On the one hand, they provide strong support for our hypothesis that enhanced Mn(III) production across these interfaces stimulates litter decomposition. On the other hand, they uncover an unexpected enzymatic response: elevated Mn(III) formation markedly amplified microbial gene expression at the oxic–anoxic interface, driving oxidative and other litter-degrading enzymes to levels of expression far exceeding those observed in the oxic zone alone. This dual outcome highlights the direct role of Mn(III) in accelerating decomposition and its capacity to reshape microbial functions that enhance enzymatic litter decomposition. Taken together, our findings underscore how reactive Mn(III) species and litter-degrading enzymes act synergistically to transform oxic–anoxic interfaces into critical hotspots of litter decomposition.

### Enhanced Mn(III) formation at oxic-anoxic interfaces drives litter decomposition

Our results reveal the transient yet impactful nature of Mn(III) formation at oxic-anoxic interfaces. These oxic-anoxic interfaces—where opposing diffusive gradients continuously supply O_2_ and Mn(II)—act as hotspots for the formation of reactive Mn(III) species and is consistent with those naturally occurring in marine waters^37,57^, marine sediments^57^, soil reactor^41^ and soil profiles^32,39,53^. The emergent Mn(III) hotspot was rather short-lived, arising after approximately 30 days and vanishing subsequently (Fig. 2). Porewater analyses (Supplementary Fig. 4) indicate that Mn(II) supply was limited by Mn oxide reduction in the anoxic zone and by diffusive transport to the interface, while concurrent changes in microbial respiration (Fig. 3a) and community composition (Fig. 3b) indicate a functional adjustment among the microbial community. Supplying more Mn(II) to the interface not only increased Mn(III) formation but also altered microbial growth and composition. These observations suggest that preferential Mn(III) formation at the oxic-interface necessitates sufficient Mn(II) supply and a functional shift of the microbial community.

Creating hotspots of Mn(III) formation at the oxic-anoxic interface by increasing Mn availability increases litter decomposition. Enhanced Mn(II) availability not only resulted in greater overall litter decomposition (Fig. 3a), but the subsequent Mn(III) formation also coincided with a dramatic increase in bacterial growth (Fig. 3b). High-resolution mass spectrometry further confirmed that Mn additions selectively depleted aromatic compound classes (e.g., condensed hydrocarbon- and tannin-like compound classes) (Supplementary Fig. 1), consistent with oxidative depolymerization of complex litter structures. These molecular patterns provide chemical evidence that enhanced Mn(III) formation at the oxic-anoxic interface initiated depolymerization of aromatic litter components, fueling microbial growth and subsequent mineralization of the resulting products to CO_2_.

Previous experiments testing the effect of Mn(II) availability on decomposition rates have provided inconsistent outcomes, showing both decreasing^31,32^ and increasing decomposition rates^14,58^. These discrepancies are likely due to excluding oxic-anoxic transitions from conventional experimental setups used to study litter decomposition^31,32^, which are often conducted at relatively low or constant moisture, ignoring the variable nature of soil redox in forest^59,60^, grassland^61,62^ and agricultural soils^43,63^. Without some degree of redox heterogeneity and associated oxic-anoxic transitions, which is expected in most soil ecosystems^42,64^, conditions favoring reactive Mn(III) species may be lacking, and hence no Mn-dependence of decomposition is found. In other words, the observed Mn-dependence of litter decomposition across various ecosystems^14,15,22^ is not merely a function of Mn availability—it is also strongly reliant on suitable soil redox conditions and associated microbially-driven enzymatic capacity for the formation and regeneration of reactive Mn(III) species driving the decomposition.

### Enzymatic drivers of Mn(III)-mediated litter decomposition

Our transcriptomic analyses confirm the enzymatic drivers of Mn(III) formation and litter decomposition. We found that increased Mn(III)-mediated decomposition coincided with a doubling of gene expression of enzymes at the oxic-anoxic interface compared to the oxic zone, which is conventionally assumed to have the highest decomposing activity. Was Mn(III) formation at the interface linked to the upregulation of specific Mn(II)-oxidizing genes? We initially expected that enhanced Mn(III) formation would be linked to enhanced expression of specific fungal peroxides that use Mn(II) as co-factor, directly oxidizing Mn(II) to reactive Mn(III) species (e.g., MnP)^65,66^. However, we found no evidence that Mn(III) formation (or decomposition) was directly linked to the individual expression of these specific enzymes in our system. Individual expression patterns of enzymes with Mn as an active center to mitigate oxidative stress (e.g., Mn-superoxide dismutase, Mn-SOD, or Mn-catalase)^67^ also showed no relationship with Mn(III) formation. Instead, Mn(III) formation was best predicted by the combined total expression of a broad suite of putatively Mn-oxidizing oxidoreductases (Supplementary Table 2) that were mostly attributed to bacteria (Supplementary Fig. 10). This broader suite of oxidoreductases includes enzymes that either directly or indirectly oxidize Mn(II) via the production of reactive intermediates^51,52,67^. The formation of reactive Mn(III) species at oxic-anoxic interfaces thus appears to be driven by a broad suite of ubiquitous oxidative enzymes in our system, rather than highly specialized Mn(III)-forming enzymes unique to litter-decomposing fungi, as originally assumed.

The pronounced effect of increased Mn(II) supply on the upregulation of gene expression at the oxic-interface was not limited to specific lignolytic oxidative enzymes (e.g., MnP, LiP, etc), but was apparent for a broad suite of oxidases, hydrolases, and transferases (Fig. 4b and Supplementary Table 2c). What might have caused this broad upregulation in gene expression? Litter decomposition is a complex process in which lignin and other biopolymers of the lignocellulose complex (e.g., (hemi)cellulose) must be degraded simultaneously^68,69^. Reactive Mn(III) intermediates penetrate the lignocellulose structures within litter^24,30^, selectively depleting tannin-like and condensed hydrocarbon-like compounds with peak Mn(III) concentrations (Supplementary Fig. 2), suggesting that reactive Mn(III) species primarily target aromatic compounds, such as lignin or tannin, during litter decomposition^24,29,70^. Thus, our study supports the notion that oxidative depolymerization of lignin structures by small reactive Mn(III) species represents the initial attack, creating sufficient porosity for larger oxidative and hydrolytic enzymes to enter. The resulting oxidative and hydrolytic depolymerization products may further support the growth and activity of the microbial community as a whole and explain the broad upregulation of other enzymes (e.g., transferases and ligases) at the oxic-anoxic interface. This explanation points to a strong interplay of Mn(III)-mediated initial attack and subsequent broader enzymatic decomposition of complex litter at oxic-anoxic interfaces.

### Synergistic effects of Mn cycling and enzymatic activity in litter decomposition

**Fig. 6:**
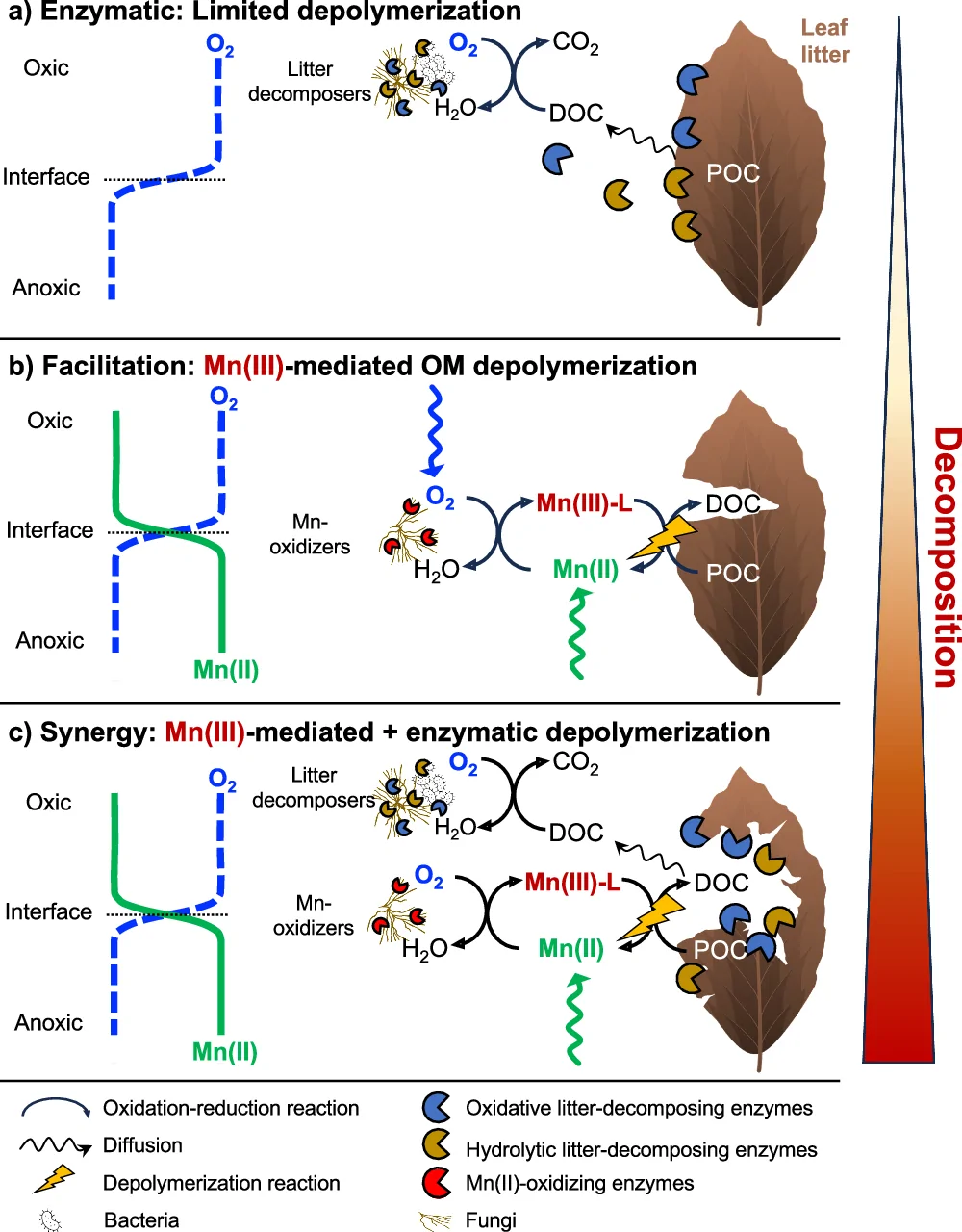
Synergistic interaction between manganese and enzymes that render oxic–anoxic interfaces hotspots of litter decomposition. Under fully oxic conditions (**a**), litter decomposition is traditionally viewed as being controlled by O_2_-dependent extracellular enzymes that catalyze the rate-limiting oxidative depolymerization of lignocellulose. This process is constrained by the large size of enzymes, which limits their ability to penetrate intact plant cell walls. At oxic–anoxic interfaces (**b**), however, opposing O_2_ and Mn(II) gradients promote the formation of reactive Mn(III)–ligand complexes by Mn(II)-oxidizing enzymes. Because these Mn(III)–ligand complexes are far smaller and more diffusible than bulky enzymes, they can more readily infiltrate intact litter and initiate oxidative depolymerization. This Mn(III)-mediated attack increases litter porosity, making structural polymers accessible to enzymatic depolymerization and thereby accelerating downstream microbial activity and overall decomposition. Our findings therefore support the view that oxic–anoxic interfaces act as hotspots of litter decomposition because they facilitate a synergistic process (**c**): reactive Mn(III) species initiate oxidative attack of intact litter, enabling oxidative and hydrolytic enzymes to further depolymerize it, together promoting faster and more complete decomposition. Note that particulate organic carbon (POC) denotes solid-phase structural components in the litter, while dissolved organic carbon (DOC) denotes the soluble products arising from POC depolymerization. The brown leaf icon is modified from Creazilla, “autumn leaf brown fall color,” public domain (CC0) (the shape was redrawn to indicate degradation). The fungi and bacteria icons were adapted from Creazilla, “tree roots” and “bacteria,” Creazilla Open License (both icons were recolored to match).

Our findings expand our conception of the molecular processes underpinning the decomposition of complex litter in soils by explicitly considering the synergies between redox-active metal catalysts, such as Mn and litter-decomposing enzymes (Fig. 6): under fully oxic conditions, where redox cycling is limited, decomposition is traditionally viewed as being controlled by extracellular enzymes secreted by fungi and bacteria (Fig. 6a). In this model, oxidative ligninolytic enzymes catalyze the rate-limiting step: the depolymerization of complex lignocellulose into smaller dissolved organic compounds that microbes can assimilate and metabolize. This oxidative step is often seen as the bottleneck of decomposition, as it is needed to enable subsequent hydrolytic enzymes to further degrade lignocellulose. However, under these conditions, decomposition is constrained by the relatively large size of enzymes, which prevents them from penetrating intact plant cell walls. Our results demonstrate that oxic–anoxic interfaces are critical for the formation of reactive intermediates, such as Mn(III) by supplying both Mn(II) and O_2_ through opposing diffusive gradients (Fig. 6b). Unlike ligninolytic enzymes—nanometer-scale globular proteins, such as manganese peroxidase (MnP, ~40–50 kDa)–Mn(III)–ligand complexes are much smaller and more highly diffusible^24^ (e.g., the Mn(III)–oxalate 1:3 complex, 319 Da). These reactive species can penetrate intact litter more readily than bulky enzymes, initiating oxidative depolymerization of lignocellulose and increasing litter porosity. This initial Mn(III)-mediated attack renders litter more accessible to subsequent enzymatic depolymerization, resulting in more comprehensive decomposition and enhances microbial activity downstream (Fig. 6c). While our study used broadleaf litter incubations, both ecosystem scale studies^15,22^ and mechanistic laboratory observations^30^ show a strong relationship between Mn availability and the decomposition of coniferous litter, suggesting that our observations are independent of litter type. Our findings therefore support the view that oxic–anoxic interfaces act as hotspots of litter decomposition more broadly because they facilitate a synergistic process: Mn(III) initiates oxidative attack of intact litter, which allows subsequent extracellular enzymatic depolymerization, together enabling faster and more complete litter decomposition.

### Implications for litter decomposition in terrestrial ecosystems

Oxidative depolymerization is a fundamental rate-limiting step in litter decomposition within soil ecosystems. Current conceptual^5,71,72^ and numerical models^73,74^ assume that this process, and by extension decomposition itself, is maximized under oxic conditions and suppressed under anoxic conditions^74^. This paradigm is grounded in the idea that oxidative enzymes require abundant O_2_ to remain active^72^, thus their activity would diminish sharply when O_2_ is limited. However, a growing body of research has begun to highlight the catalytic role of redox-active metal intermediates, such as Mn, Fe, and Co^8,10,30^, particularly in soils subject to dynamic redox transitions^9,75^. Our results challenge the traditional view that oxidative enzymatic decomposition is inherently strongest within oxic soil spaces. Instead, we show that the synergistic interplay between redox-active metals and microbial enzymes generates pronounced hotspots of oxidative decomposition at oxic–anoxic interfaces. These interfaces are rather widespread in soils: they arise in forest floor litter layers^76^, soil aggregates^42,77^, rhizospheres^78^, and capillary fringes^79^ and occur across ecosystems as diverse as agricultural^43^, grassland^62^, forest^40,80^, and wetland soils^81^. By demonstrating that oxic–anoxic interfaces act as critical regulators of litter decomposition, our findings highlight the significance of these microenvironments as underappreciated “ecosystem control points”^82^ that govern rates of litter decomposition in many soils. Most importantly, recognizing that decomposition is facilitated by the coupled action of metal-mediated and enzyme-mediated pathways requires a fundamental shift in how decomposition is conceptualized in ecosystem-scale models. The conventional equation of oxic conditions with “fast” decomposition and anoxic conditions with “slow” decomposition^74^ is no longer tenable. To improve predictions of litter decomposition rates under changing environmental conditions, ecosystem models must explicitly account for the catalytic potential of oxic–anoxic boundaries and associated synergistic interactions between redox-active metals and microbial enzymes. Incorporating these mechanisms will be critical for capturing the spatial and temporal complexity of litter decomposition in terrestrial ecosystems.

## Methods

### Diffusion reactors

To establish a stable oxic-anoxic interface in soil across which we could manipulate Mn availability, we adopted an established design of diffusion reactors^41^. Specifically, to accommodate increased sampling volume, the modified reactors were constructed with a larger polypropylene shell (20 × 11 × 3 cm) with openings at the top to allow oxygen to diffuse within the soil matrix (Supplementary Fig. 11). An inlet at the bottom of the reactor allowed us to regulate the water table height and establish stable oxic-anoxic interfaces. To determine changes in dissolved Mn concentrations across the soil column, we installed MicroRhizon pore water samplers (Rhizosphere Research Products, Wageningen, Netherlands) in 5 mm increments across the oxic-anoxic interface for periodic sampling. Reactors could be periodically sealed airtight to measure CO_2_ production as a quantification of overall litter oxidation during the experiment.

### Reactor experiment

To follow the decomposition of naturally occurring complex litter while manipulating Mn availability, all reactors were packed with a 5:95 wt% litter to sand ratio. Fresh deciduous litter composed of red oak (*Quercus rubra*), red maple (*Acer rubrum*), paper birch (*Betula papyrifera*), striped maple (*Acer pennsylvanicum*), and white ash (*Fraxinus americana*) leaves were collected during the Fall of 2020 at an established and well-characterized field site^53,83^ (Harvard Forest, Petersham, MA). Litter was collected at this site because previous work provided a thorough assessment of litter quality and Mn speciation and demonstrated a strong relationship between Mn(III) and potential for litter decomposition^53,83,84^. Litter was gently washed with tap water of loose soil and debris, air dried, and homogenized by gently grinding to a particle size of less than 2 mm. To provide the necessary porosity for sufficient fungal growth^40^, and measurable diffusive gradients, the litter was then mixed at a 5:95 wt% ratio with acid-washed quartz sand (sieved to <0.3 mm) (Fisher Scientific, Hampton, NH). This sand/litter mixture represented the upper 2/3 of the overall reactor.

To manipulate Mn(II) supply towards the oxic-anoxic interface, we added Mn oxides to the bottom layer of the reactors. We established three different treatments: a *“control”* in which the bottom layer consisted of sand and litter (at the mixing ratio indicated above), containing only the Mn naturally contained in the litter; as well as *“low Mn”* and *“high Mn”* treatments, in which the sand—prior to packing, was coated with Mn(III,IV) oxides to a level reflecting soil Mn concentrations at the site; and a “high Mn” treatment, in which the sand was coated with Mn(III,IV) oxides to represent commonly observed ranges of total Mn concentrations in forest litter and soil (400 and 4000 µg/g, respectively)^15,85^. Mn(III/IV) oxides were composed of acid birnessite synthesized according to an established protocol^56^. A slurry of Mn oxides was coated onto acid-washed sand to achieve the desired concentration. The Mn oxide-coated sand was air-dried for 96 h and mixed with litter at the aforementioned ratio. The resulting mixture was then filled into the bottom of the reactor. The bottom layer of the “high” and “low” Mn treatments received the Mn oxide-coated sand/litter mixture, while the “control” treatment received only the sand/litter mixture without additional Mn oxides. This bottom layer represented 1/3 of the overall mass in the reactor. This bottom layer containing Mn oxides was added to the reactors first, followed by the addition of the layer only containing the litter/sand mixture. Both layers were partially wetted before packing to avoid separation of the lighter litter and the heavier sand. The resulting “soil” column within the reactor had an approximate height of 75 mm.

To create a stable water table, an inlet at the bottom of the reactor was connected to a reservoir containing an artificial groundwater soil solution (3.8 mM KCl, 3.5 mM MgSO_4_, 3.7 mM CaSO_4_·2H_2_O, 0.009 mM KH_2_PO_4_, 3.7 mM NaHCO_3_, 0.064 mM KNO_3_, adjusted to a pH of 5.5)^40,41^, establishing a hydrostatic equilibrium between the two. The water table was maintained 15 mm below the top of the soil surface. The oxic-anoxic boundary was defined by periodically profiling the dissolved oxygen concentrations in select reactors using microelectrodes^41^. Based on water table position and dissolved oxygen profiles, we designated 0–10 mm below the soil surface as the oxic zone, 10–15 mm as the oxic-anoxic interface, and 15–50 mm as the anoxic interface. Depths of 50–75 mm below the surface marked the bottom layer receiving Mn(III/IV) additions. Reactors were incubated in the dark at 23 °C. Quadruplicate reactors for each treatment (control as well as Low and High Mn) were set up for destructive harvest at three time points.

### CO_2_ and dissolved Mn measurements

CO_2_ measurements were conducted throughout the experiment to quantify differences in CO_2_ production due to treatment effects. To quantify CO_2_ production over time, openings on top of the reactors were sealed with silicon stoppers for 30 min before measurement. Headspace air was sampled through the septa using an airtight syringe and quantified on a Quantek infrared gas analyzer (IRGA) model 906^86^ (Quantek Instrument Inc., Grafton, MA). Measured concentrations were converted to CO_2_ measurements for each time point to calculate cumulative CO_2_ production over the course of the experiment. Sealants were then removed after measurement. Duplicate gas sampling for each reactor occurred weekly for the duration of the experiment. To quantify Mn in the reactor solution, pore water was collected from MicroRhizon samplers. Depth-resolved collection of pore water samples through rhizon samplers occurred every 3 days during the first 30 days of the incubation, and then decreased in frequency until the end of the experiment. Pore water samples were immediately acidified and subsequently analyses by ICP-MS (Shimadzu ICPMS-2030, Kyoto, Japan).

### Reactor sampling

Based on monitoring of CO_2_ production and pore water Mn chemistry, the three sets of reactors were destructively harvested after 7, 30, and 90 days to further determine Mn and litter transformations, as well as microbial dynamics. Reactors were harvested in an anoxic atmosphere (97% N_2_ and 3% H_2_) within an anaerobic chamber (Coy Laboratory Products Inc., Ann Arbor, MI) to minimize sample oxidation due to exposure to ambient air. The soil profile was then sampled at the 0–10 mm (oxic), 10–15 (interface), 15–50 (anoxic), and 50–75 mm (Mn treatment zone) depths. Reactor samples were carefully drained of liquid first before being dismantled. Samples marked for sequential extractions, solid-phase characterization, and microbial analysis were subdivided and then removed from the anaerobic chamber. Samples were immediately flash frozen with liquid nitrogen. Samples that were designated for geochemical assays were then freeze-dried using a FreeZone lyophilizer (Labconco, Kansas City, MO) for 72 h before being stored in an anaerobic chamber until further processing. Samples designated for microbial assays were immediately flash frozen in liquid nitrogen and stored at −80 °C.

### Mn characterization

Mn transformations in general and Mn(III) formation in particular were tracked through a combination of selective wet-chemical extractions and Mn K-edge XANES. Reactive Mn phases and total abundance of Mn phases of different crystallinity were determined using sequential extractions, following a modified protocol adopted from Jones et al.^53^. Sequential extractions were done using a sequence of ultrapure H_2_O (representing water-soluble Mn), ammonium acetate (representing exchangeable Mn^87^), sodium pyrophosphate (representing organically complexed Mn), and dithionite-HCl (representing total reducible Mn^87^) as the extractants. This extraction sequence allowed us to quantify not only potentially bioavailable Mn (H_2_O and ammonium acetate), but also crystalline and total Mn (dithionite-HCl) within the soil. Sodium pyrophosphate was used due to its high affinity for Mn(III)^41^, allowing us to quantify Mn(III) after accounting for water-soluble and exchangeable Mn. Approximately 0.33 g of sample was extracted with 10 mL Milli-Q ultrapure H_2_O with shaking for 1 h (120 rpm), followed by centrifugation for 1 h (4000 × *g* RCF) and filtration through 0.22 µm syringe filters. The extraction supernatant was collected, and the remaining pellet was resuspended with 10 mL of 1 M ammonium acetate (pH = 7), shaken for 4 h, centrifuged for 1 h, and then filtered again. The ammonium acetate supernatant was removed, and the remaining pellet was resuspended with 10 mL of 0.1 M sodium pyrophosphate (pH = 10), shaken for 1 h before centrifugation for 1 h, and filtering and collecting the supernatant. The remaining pellet was resuspended with 10 mL of 0.05 M sodium dithionite, with 16 h of shaking before centrifugation for 1 h and filtering. The remaining pellet was then washed with 0.05 M HCl, shaken for 1 h, centrifuged for 1 h, before filtering and combining with the dithionite extract. Extracts were diluted and immediately analyzed by ICP-MS (Shimadzu ICPMS-2030, Kyoto, Japan).

To determine the average Mn oxidation state of the solid samples, Mn K-edge XANES spectra of all samples were collected at the soft X-ray micro-characterization beamline at the Canadian Light Source (Saskatoon, Saskatchewan, Canada). Homogenized samples from quadruplicate reactors were mounted on carbon tape, sealed with X-ray transparent Kapton tape, and spectra were collected around the Mn K-edge (6539 eV) at a range of 6510–6700 eV with 0.3 eV step sizes. Spectra were calibrated, background collected and normalized, and then linear combination fitting in Athena under the Demeter package was used to quantify the relative abundance of Mn(II), Mn(III), and Mn(IV)^88,89^ and to determine the average Mn oxidation state. Changes in the relative abundance of the Mn oxidation states were normalized to total extractable Mn to obtain absolute concentrations of Mn(II), Mn(III), and Mn(IV).

### Litter decomposition

To quantify changes in extractable organic compounds, we utilized Fourier transform ion cyclotron resonance mass spectrometry (FT-ICR MS). Approximately 100 mg of freeze-dried soil from each sample was extracted with 2 mL of 80:20 methanol:water (v/v)^90^ using HPLC-grade solvents. Samples were sonicated for 2 h at room temperature, then centrifuged at 5000 rpm for 10 min. The supernatant was collected and purified using solid phase extraction (SPE) cartridges (Agilent Bond Elut PPL, 100 mg) to remove salts and other contaminants. Extracts were eluted with 1.5 mL of HPLC-grade methanol and stored at −20 °C until analysis.

High-resolution mass spectra were collected using a 9.4-Tesla Bruker SolariX FT-ICR mass spectrometer at the University of Arizona (Tucson, AZ). Samples were directly infused into the instrument at 3 μL/min using a syringe pump. The FT-ICR MS was equipped with an electrospray ionization (ESI) source operated in negative mode with needle voltage set to −4.2 kV. Ion accumulation time was optimized at 0.08 s, and data were collected from 100 to 900 *m/z* with a resolution of 300,000 at *m/z* 400. A total of 144 scans were co-added for each sample and internally calibrated using organic matter homologous series separated by 14 Da (–CH_2_ groups). Mass measurement accuracy was <1 ppm across the measured *m/*z range.

Raw spectra were processed using Bruker DataAnalysis (version 4.2) with the FTMS peak picker module, applying a signal-to-noise ratio threshold of 7 and a minimum absolute intensity of 100. Molecular formulas were assigned using Formularity^91^, allowing for C, H, O, N, S, and P, with mass measurement error <1 ppm. Afterwards, data was preprocessed by removing all compounds with detection in less than half of the replicates within each unique treatment to focus on differences within the predominant compounds making up the samples. Peaks were also filtered to only include masses between 300 and 900 *m/z*, which ultimately retained a total of 3599 peaks (from the initial 25,627) for analysis. We then analyzed compounds using MetaboDirect^92^ to determine the relative abundance of amino sugars, lignin, tannin, proteins, lipids, condensed hydrocarbons, and unsaturated hydrocarbons. Compound classes were assigned based on H/C and O/C ratios following standard van Krevelen regions.

### RNA extractions and purification

Total RNA was extracted from 2 g flash-frozen soils using the RNeasy Power Soil Total RNA kit using the manufacturer's instructions (Qiagen). RNA was quantified using Qubit and quality measured using a BioAnalyzer 2100 (Agilent Technologies, Santa Clara, CA, USA). Genomics service and sequencing was performed at the Genomics Resource Laboratory (University of Massachusetts Amherst, MA, USA). The RNA was quantified using Qubit RNA HS assay (Life Technologies Corp), and quality was assessed on a 2100 Bioanalyzer using RNA 6000 Nano or Pico Assay (Agilent Technologies Inc). Total RNA (~100 ng) was used for RNAseq library preparation using the NEBNext UltraII Directional RNA Library Prep Kit for Illumina (New England Biolabs) following manufacturer instructions. The libraries were quantified using Qubit DNA HS assay (Life Technologies Corp), and 2100 Bioanalyzer (Agilent Technologies Inc). Libraries were sequenced on the Illumina NextSeq 500 platform using NextSeq 500/550 High Output v2.5 kit (300 cycles) with 151 bp paired-end sequencing chemistry. The RNA quality assessment, library preparation and Illumina Next-Generation Sequencing was performed at Genomics Resource Laboratory, Institute for Applied Life Sciences, University of Massachusetts Amherst, MA.

### Sequence data processing and annotation

We used metatranscriptomics to quantify shifts in microbial enzymatic gene expression and their link to Mn(II) oxidation and Mn(III) formation as a function of treatment and sampling location. Initial data curation based on base scores and adapter trimming were performed in BaseSpace (Illumina, San Diego, CA, United States), where samples were demultiplexed based on barcode prefix. Sequenced libraries were quality controlled using FastQC (v. 0.11.5)^93^. Filtered reads were then trimmed via adapter removal with Trimmomatic^94^ using *Q* < 33 (v. 0.32) using ILLUMINA TrueSeq3-SE adapters and dropping reads below a minimum length of 70 bases. The paired forward and reverse reads were then merged using Pandaseq^95^ (v. 2.11). Merged sequence reads were filtered to exclude ribosomal RNA (rRNA) using sortmeRNA (v. 3.0.2)^96^, and referenced against eukaryote 18S and 20S, bacteria 16S and 23S, and archaea 16S and 23S ribosomal RNA genes based on the SILVA databases^97^. Compiled non-rRNA sequences were then queried using blastx within DIAMOND (v. 2.0.4)^98^ referencing the NCBI-nr database for both taxonomic and functional assignment. Annotated sequences were mapped to both taxonomic classifications using NCBI taxonomy and functional classification using EC numbers based on KEGG analysis in MEGAN (parameters: minimum bit score, 50; top percent 10; minimum support, 0.05; LCA, Naive) (v. 6.23.2)^99^. Additional annotation using the CAZy database^100^ using HMMER^101^ was used to focus on enzymes associated with the degradation of litter compounds like cellulose, hemicellulose, and lignin.

### Functional and taxonomic analysis of RNA transcripts

The relative abundance of putative Mn-oxidizing enzymes in our samples were normalized using total mRNA reads per individual sample. Treatments were then averaged (*n* = 4) to determine changes in the relative abundance of genes related to Mn oxidation between treatments across the oxygen gradient. To determine the microbial response to enhanced Mn availability, target genes associated with enzymes that oxidize Mn (Supplementary Table 4) were quantified. These enzymes contain those that facilitate direct enzymatic oxidation, including multicopper oxidases and peroxidases, as well as enzymes producing reactive oxygen species to facilitate indirect Mn(II) oxidation.

To determine the microbes associated with Mn oxidation, EC assignments via KEGG analysis in MEGAN6 were linked with transcripts’ taxonomic annotations. We then examined changes in the relative abundance of all putative Mn-oxidizing enzymes associated with bacteria versus fungi, as well as enzyme categories of oxidoreductases, hydrolases, transferases, translocases, isomerases, ligases, and lyases. Analyses to determine differences in gene expression libraries of our samples were performed in RStudio using the edgeR package^102^.

### Statistical analysis

To compare differences both between treatments and the spatial dynamics of our Mn, C, and metatranscriptome analysis, we conducted 2-way ANOVAs using the Tukey test with a significance threshold of *p* < 0.05, accounting for interactions. For CO_2_ measurements, a model respiration curve fitted to the data was created for each treatment, and then the models were compared to each other using an F-statistic to determine if treatments were statistically different from each other. For comparisons between two variables using linear correlations, an F-test was conducted with a significance threshold of *p* < 0.05. Correlation analyses were done using Pearson’s correlation coefficient. Microbial abundance via qPCR was analyzed using 3-way ANOVA, with treatment, sampling location, and timepoint as the variables. ANOVAs were conducted using OriginPro 9.95 (OriginLab). For determining expression profiles were different among treatments, we conducted an ANOSIM analysis on a Bray-Curtis dissimilarity ran using transformed normalized gene expression profiles (log (*x* + 1)) to minimize the effect of genes with 0 counts. Pair-wise comparisons of combinations of treatment and sampling location were used for ANOSIM analysis to determine statistical significance (*p* < 0.05).

### Reporting summary

Further information on research design is available in the Nature Portfolio Reporting Summary linked to this article.

## Supplementary information

Supplementary Information

Reporting Summary

Transparent Peer Review File

## Data Availability

Code and data from this study are available on GitHub at https://github.com/mkeiluweit/manganese. Supporting information include Mn wet-chemical extraction data, pore water Mn measurements, Mn(III) quantification, qPCR data, FT-ICR MS data, FTIR measurements, ANOSIM pair-wise comparisons between gene expression profiles, regression fits, gene expression profiles, and a table of selected Mn-oxidizing enzymes.
