# Supplementary material for: Synergy between reactive manganese species and enzymes drives litter decomposition hotspots
Source: Nat Commun. 2026 Jul 25;17:10355. doi: 10.1038/s41467-026-75920-x (PMC13623943; doi:10.1038/s41467-026-75920-x)
Supplement: Supplementary file 1 [file 41467_2026_75920_MOESM1_ESM.pdf]

- Supplementary Information -

Synergy between reactive manganese species and enzymes drives litter decomposition hotspots

Nathan A. Chin[1], Luiz A. Domeignoz-Horta[2], Xiao Jun A. Liu[3], Morris E. Jones[4], Ravi Ranjan[5], Malak Tfaily[6], Kristen M. DeAngelis[7], and Marco Keiluweit[1,]*

[1] Institute of Earth Surface Dynamics, Faculty of Geosciences and Environment, University of Lausanne, Lausanne, 1015, Switzerland
[2] French National Institute for Agriculture, Food, and Environment, Paris, 75338, France
[3] Department of Biological Sciences, Louisiana State University, Baton Rouge, 70803, USA
[4] College of Health and Natural Sciences, Franklin Pierce University, Rindge, NH, 03461
[5] Genomics Resource Laboratory, Institute for Applied Life Sciences, University of Massachusetts, Amherst, MA, 01003, USA
[6] Department of Environmental Science, University of Arizona, Tucson, Arizona, 85721, USA
[7] Microbiology Department, University of Massachusetts, Amherst, MA, 01003, USA

*Corresponding author: Prof. Dr. Marco Keiluweit, marco.keiluweit@unil.ch

Supplementary Table 1. Matrix of pair-wise comparisons of gene expression in reactors for differences between treatment and location

Treatment/ Location	Control						Low Mn						High Mn					
	Oxic		OAI		Anoxic		Oxic		OAI		Anoxic		Oxic		OAI		Anoxic	
	Similarity (R)	p-value	Similarity (R)	p-value	Similarity (R)	p-value	Similarity (R)	p-value	Similarity (R)	p-value	R	p-value	Similarity (R)	p-value	Similarity (R)	p-value	Similarity (R)	p-value
Control																		
Oxic	-	-	-	-	-	-	-	-	-	-	-	-	-	-	-	-	-	-
OAI	0.438	0.57	-	-	-	-	-	-	-	-	-	-	-	-	-	-	-	-
Anoxic	**1.00**	**0.029***	**0.552**	**0.029***	-	-	-	-	-	-	-	-	-	-	-	-	-	-
Low Mn																		
Oxic	0.01	0.31	0.521	0.11	**0.979**	**0.029***	-	-	-	-	-	-	-	-	-	-	-	-
OAI	**0.51**	**0.029***	0.74	0.57	**1.00**	**0.029***	0.021	0.60	-	-	-	-	-	-	-	-	-	-
Anoxic	**0.948**	**0.029***	**0.333**	**0.029***	-0.063	0.86	**0.906**	**0.029***	**1.00**	**0.029***	-	-	-	-	-	-	-	-
High Mn																		
Oxic	**0.481**	**0.029***	**0.333**	**0.029***	**0.889**	**0.029***	0.185	0.086	**0.407**	**0.029***	**0.759**	**0.029***	-	-	-	-	-	-
OAI	**0.906**	**0.029***	**0.938**	**0.029***	**1.00**	**0.029***	**0.594**	**0.029***	0.510	0.14	**1.00**	**0.029***	0.370	0.51	-	-	-	-
Anoxic	**1.00**	**0.029***	**0.521**	**0.029***	**0.469**	**0.029***	**0.948**	**0.029***	**1.00**	**0.029***	**0.156**	**0.029***	**0.741**	**0.029***	1.00	0.057	-	-

ANOSIM statistical test was conducted on a Bray-Curtis dissimilarity analysis on log(x+1) transformed values of normalized gene expression profiles. The Similarity value denotes the R statistic (range -1 to 1), wherein a value of 1 indicates that the gene expression profiles are completely distinct (dissimilar) whereas R values close to 0 indicate similar profiles. Statistically significant comparisons are in bold and denoted with (*) ($p < 0.05$) between the replicates of different reactor treatments and locations (n=4).

Supplementary Table 2. Linear correlation statistics between quantifications of extraction pools of Mn and Mn(III) to the relative expression of different EC enzyme classes and a curated list of putative Mn-oxidizing enzymes at each sampling location

a) All

	Water-Extractable Mn		*Ammonium Acetate – Extractable Mn*		*Dithionite Extractable Mn*		*Pyrophosphate Extractable Mn*		*Total Mn(III)*	
Enzyme Categories	R^2	*p-value*	R^2	*p-value*	R^2	*p-value*	R^2	*p-value*	R^2	*p-value*
E.C. 1 Oxidoreductases	0.0008	0.32	0.071	0.067^	**0.102**	**0.034***	**0.187**	**0.0055***	**0.198**	**0.0043***
E.C. 2 Transferases	0.011	0.25	**0.131**	**0.019***	**0.175**	**0.0073***	**0.274**	**0.00075***	**0.287**	**0.0005***
E.C. 3 Hydrolases	0.00121	0.32	0.067	0.073^	**0.130**	**0.019***	**0.153**	**0.012***	**0.167**	**0.0085***
E.C. 4 Lyases	-0.0046	0.36	0.0077	0.27	0.043	0.12	0.048	0.108	0.058	0.087^
E.C. 5 Isomerases	0.00055	0.32	**0.089**	**0.046***	**0.167**	**0.0086***	**0.185**	**0.0058***	**0.197**	**0.0044***
E.C. 6 Ligases	-0.0065	0.38	0.056	0.092^	**0.108**	**0.031***	**0.150**	**0.012***	**0.159**	**0.010***
E.C. 7 Translocases	-0.022	0.60	0.017	0.22	0.067	0.073^	**0.116**	**0.026***	**0.121**	**0.023***
Mn-Oxidizing Enzymes	0.044	0.12	**0.154**	**0.011***	**0.149**	**0.013***	**0.196**	**0.0046***	**0.205**	**0.0037***

b) Oxic

Enzyme Categories	*Water-Extractable Mn*		*Ammonium Acetate – Extractable Mn*		*Dithionite Extractable Mn*		*Pyrophosphate Extractable Mn*		*Total Mn(III)*	
	R^2	*p-value*	R^2	*p-value*	R^2	*p-value*	R^2	*p-value*	R^2	*p-value*
E.C. 1 Oxidoreductases	-0.029	0.42	-0.082	0.63	-0.083	0.64	-0.061	0.53	-0.071	0.58
E.C. 2 Transferases	-0.072	0.58	-0.064	0.54	-0.074	0.59	-0.034	0.43	-0.049	0.48
E.C. 3 Hydrolases	-0.110	0.93	-0.038	0.44	-0.080	0.62	0.014	0.31	-0.015	0.38
E.C. 4 Lyases	-0.007	0.36	-0.111	0.99	-0.108	0.89	-0.104	0.82	-0.109	0.91
E.C. 5 Isomerases	0.014	0.31	-0.108	0.87	-0.083	0.64	-0.111	0.95	-0.110	0.94
E.C. 6 Ligases	-0.057	0.51	-0.091	0.69	-0.092	0.70	-0.066	0.55	-0.078	0.61
E.C. 7 Translocases	-0.109	0.91	0.169	0.12	0.175	0.11	0.189	0.10	0.182	0.11
Mn-Oxidizing Enzymes	0.147	0.13	0.050	0.25	0.046	0.25	0.024	0.29	0.036	0.27

c) Oxic-Anoxic Interface

	Water-Extractable Mn		*Ammonium Acetate – Extractable Mn*		*Dithionite Extractable Mn*		*Pyrophosphate Extractable Mn*		*Total Mn(III)*	
Enzyme Categories	R^2	*p-value*	R^2	*p-value*	R^2	*p-value*	R^2	*p-value*	R^2	*p-value*
E.C. 1 Oxidoreductases	**0.588**	**0.0022***	**0.293**	**0.040***	0.131	0.13	0.243	0.059^	0.249	0.056^
E.C. 2 Transferases	**0.532**	**0.0043***	**0.330**	**0.030***	0.176	0.097^	**0.280**	**0.045***	**0.287**	**0.042***
E.C. 3 Hydrolases	**0.397**	**0.017***	0.127	0.14	0.093	0.18	0.086	0.18	0.097	0.17
E.C. 4 Lyases	0.389	0.18	0.033	0.27	0.017	0.30	0.002	0.34	0.011	0.31
E.C. 5 Isomerases	**0.307**	**0.036***	0.186	0.091^	0.178	0.096^	0.172	0.10	0.178	0.096^
E.C. 6 Ligases	0.251	0.056^	0.094	0.17	0.061	0.22	0.081	0.19	0.086	0.18
E.C. 7 Translocases	0.155	0.11	0.040	0.26	0.014	0.31	0.040	0.25	0.041	0.25
Mn-Oxidizing Enzymes	**0.633**	**0.0012***	**0.411**	**0.015***	**0.265**	**0.050***	**0.358**	**0.023***	**0.368**	**0.022***

d) Anoxic

	Water-Extractable Mn		*Ammonium Acetate – Extractable Mn*		*Dithionite Extractable Mn*		*Pyrophosphate Extractable Mn*		*Total Mn(III)*	
Enzyme Categories	R^2	*p-value*	R^2	*p-value*	R^2	*p-value*	R^2	*p-value*	R^2	*p-value*
E.C. 1 Oxidoreductases	-0.099	0.91	-0.086	0.72	-0.084	0.71	-0.076	0.65	-0.084	0.71
E.C. 2 Transferases	-0.024	0.41	-0.023	0.40	-0.045	0.49	-0.044	0.48	-0.031	0.43
E.C. 3 Hydrolases	-0.0001	0.34	-0.013	0.38	-0.025	0.41	-0.038	0.46	-0.028	0.42
E.C. 4 Lyases	-0.099	0.93	-0.095	0.83	-0.098	0.89	-0.090	0.76	-0.094	0.82
E.C. 5 Isomerases	-0.079	0.67	-0.043	0.48	-0.075	0.64	-0.057	0.54	-0.056	0.53
E.C. 6 Ligases	-0.087	0.74	-0.092	0.79	-0.087	0.74	-0.085	0.72	-0.085	0.72
E.C. 7 Translocases	-0.065	0.58	-0.036	0.45	-0.040	0.47	-0.024	0.41	-0.032	0.44
Mn-Oxidizing Enzymes	-0.100	1.00	-0.099	0.94	-0.098	0.90	-0.096	0.86	-0.099	0.91

Linear regression correlations between the gene expression of each enzyme class (normalized to total mRNA) and putative Mn-cycling enzymes to the quantity of each Mn extraction pool. R^2 and p-values are listed for each individual sampling zone – a) aggregated across all sampling zones, b) the oxic, c) oxic-anoxic interface, and d) anoxic zone. Correlations bolded with (*) notation indicates statistically significant correlations ($p < 0.05$) while marginally significant correlations ($p < 0.10$) are noted with (^) for samples (n=4). Significance was determined using the F statistic for the drawn linear correlations.

Supplementary Table 3. Linear correlation statistics between cumulative CO_2 production and the relative expression of different EC enzyme classes and a curated list of putative Mn-oxidizing enzymes at each sampling location

	All		*Oxic*		*Oxic-Anoxic Interface*		*Anoxic*	
Enzyme class correlation to cumulative CO_2 production	R^2	*p-value*	R^2	*p-value*	R^2	*p-value*	R^2	*p-value*
E.C. 1 Oxidoreductases	0.00045	0.32	-0.046	0.47	**0.276**	**0.046***	-0.090	0.77
E.C. 2 Transferases	0.0212	0.20	0.131	0.15	**0.394**	**0.017***	-0.005	0.35
E.C. 3 Hydrolases	0.0108	0.25	**0.328**	**0.038***	0.251	0.056^	0.101	0.17
E.C. 4 Lyases	-0.028	0.79	0.122	0.16	0.095	0.17	-0.100	1.00
E.C. 5 Isomerases	0.0231	0.19	-0.106	0.85	0.206	0.078^	0.121	0.14
E.C. 6 Ligases	0.0201	0.20	0.100	0.18	**0.320**	**0.032***	-0.099	0.94
E.C. 7 Translocases	0.0231	0.19	-0.050	0.49	0.244	0.059^	-0.100	0.97
Mn-Oxidizing Enzymes	0.0713	0.07^	-0.027	0.41	**0.337**	**0.028***	-0.099	0.94

Linear regression correlations between the gene expression of each enzyme class (normalized to total mRNA) and putative Mn-cycling enzymes to cumulative CO_2 production measured by the end of the incubation period. Adjusted R^2 and p-values are listed for the reactors as a whole and each individual sampling zone. Correlations bolded with (*) notation indicates statistically significant correlations ($p < 0.05$) while marginally significant correlations ($p < 0.10$) are noted with (^) for samples (n=4). Significance was determined using the F statistic for the drawn linear correlations.

Supplementary Table 4. Curated list of enzymes with putative roles in Mn oxidation

Enzyme Commission Number	*Enzyme Name*	*Notes*
1.1.3.10	Pyranose Oxidase	Produces H_2O_2 during reaction
1.1.3.11	L-Sorbose Oxidase	Produces H_2O_2 during reaction
1.1.3.12	Pyridoxine 4-Oxidase	Produces H_2O_2 during reaction
1.1.3.13	Alcohol Oxidase	Produces H_2O_2 during reaction
1.1.3.15	(S)-2-Hydroxy-Acid Oxidase	Produces H_2O_2 during reaction
1.1.3.16	Ecdysone Oxidase	Produces H_2O_2 during reaction
1.1.3.17	Choline Oxidase	Produces H_2O_2 during reaction
1.1.3.18	Secondary-Alcohol Oxidase	Produces H_2O_2 during reaction
1.1.3.19	4-Hydroxymandelate Oxidase (Decarboxylating)	Produces H_2O_2 during reaction
1.1.3.20	Long-Chain-Alcohol Oxidase	Produces H_2O_2 during reaction
1.1.3.21	Glycerol-3-Phosphate Oxidase	Produces H_2O_2 during reaction
1.1.3.23	Thiamine Oxidase	Produces H_2O_2 during reaction
1.1.3.27	Hydroxyphytanate Oxidase	Produces H_2O_2 during reaction
1.1.3.29	N-Acylhexosamine Oxidase	Produces H_2O_2 during reaction
1.1.3.30	Polyvinyl-Alcohol Oxidase	Produces H_2O_2 during reaction
1.1.3.37	D-Arabinono-1,4-Lactone Oxidase	Produces H_2O_2 during reaction
1.1.3.38	Vanillyl-Alcohol Oxidase	Produces H_2O_2 during reaction
1.1.3.39	Nucleoside Oxidase (H_2O_2-Forming)	Produces H_2O_2 during reaction
1.1.3.4	Glucose Oxidase	Produces H_2O_2 during reaction
1.1.3.40	D-Mannitol Oxidase	Produces H_2O_2 during reaction
1.1.3.41	Alditol Oxidase	Produces H_2O_2 during reaction
1.1.3.42	Prosolanapyrone-II Oxidase	Produces H_2O_2 during reaction
1.1.3.43	Paromamine 6'-Oxidase	Produces H_2O_2 during reaction
1.1.3.44	6'''-Hydroxyneomycin C Oxidase	Produces H_2O_2 during reaction
1.1.3.45	Aclacinomycin-N Oxidase	Produces H_2O_2 during reaction
1.1.3.46	4-Hydroxymandelate Oxidase	Produces H_2O_2 during reaction
1.1.3.47	5-(Hydroxymethyl)Furfural Oxidase	Produces H_2O_2 during reaction
1.1.3.48	3-Deoxy-Alpha-D-Manno-Octulosonate 8-Oxidase	Produces H_2O_2 during reaction
1.1.3.49	(R)-Mandelonitrile Oxidase	Produces H_2O_2 during reaction
1.1.3.5	Hexose Oxidase	Produces H_2O_2 during reaction
1.1.3.6	Cholesterol Oxidase	Produces H_2O_2 during reaction
1.1.3.7	Aryl-Alcohol Oxidase	Produces H_2O_2 during reaction
1.1.3.8	L-Gulonolactone Oxidase	Produces H_2O_2 during reaction
1.1.3.9	Galactose Oxidase	Produces H_2O_2 during reaction
1.10.3.16	Dihydrophenazinedicarboxylate Synthase	Produces H_2O_2 during reaction
1.10.3.17	Superoxide Oxidase	Produces superoxide

1.10.3.5	3-Hydroxyanthranilate Oxidase	Produces H_2O_2 during reaction
1.10.3.6	Rifamycin-B Oxidase	Produces H_2O_2 during reaction
1.11.1.1	NADH Peroxidase	H_2O_2 consuming
1.11.1.10	Chlorine Peroxidase	H_2O_2 consuming
1.11.1.11	L-Ascorbate Peroxidase	H_2O_2 consuming
1.11.1.13	Manganese Peroxidase	H_2O_2 consuming
1.11.1.14	Lignin Peroxidase	H_2O_2 consuming
1.11.1.16	Versatile Peroxidase	Uncategorized peroxidases
1.11.1.17	Glutathione Amide-Dependent Peroxidase	H_2O_2 consuming
1.11.1.18	Bromide Peroxidase	H_2O_2 consuming
1.11.1.19	Dye Decolorizing Peroxidase	H_2O_2 consuming
1.11.1.2	NADPH Peroxidase	H_2O_2 consuming
1.11.1.21	Catalase Peroxidase	H_2O_2 consuming
1.11.1.23	(S)-2-Hydroxypropylphosphonic Acid Epoxidase	H_2O_2 consuming
1.11.1.3	Fatty-Acid Peroxidase	H_2O_2 consuming
1.11.1.5	Cytochrome-C Peroxidase	H_2O_2 consuming
1.11.1.6	Catalase	H_2O_2 consuming
1.11.1.7	Peroxidase	H_2O_2 consuming
1.11.1.8	Iodide Peroxidase	H_2O_2 consuming
1.11.1.9	Glutathione Peroxidase	H_2O_2 consuming
1.11.2.1	Unspecific Peroxygenase	H_2O_2 consuming, ROH producing
1.11.2.2	Myeloperoxidase	H_2O_2 consuming
1.11.2.4	Fatty-Acid Peroxygenase	H_2O_2 consuming
1.11.2.5	3-Methyl-L-Tyrosine Peroxygenase	H_2O_2 consuming
1.11.2.6	L-Tyrosine Peroxygenase	H_2O_2 consuming
1.15.1.2	Superoxide Reductase	Produces superoxide
1.16.3.3	Manganese Oxidase	Directly oxidizes Mn
1.16.3.4	Cuproxidase	CueO
1.16.9.1	Xanthine Oxidase	Produces H_2O_2 during reaction
1.17.3.2	6-Hydroxynicotinate Dehydrogenase	Produces H_2O_2 during reaction
1.17.3.3	Aldehyde Oxidase	Produces H_2O_2 during reaction
1.2.3.1	Abscisic-Aldehyde Oxidase	Produces H_2O_2 during reaction
1.2.3.14	(Methyl)Glyoxal Oxidase	Produces H_2O_2 during reaction
1.2.3.15	Pyruvate Oxidase	Produces H_2O_2 during reaction
1.2.3.3	Oxalate Oxidase	Produces H_2O_2 during reaction
1.2.3.4	Glyoxylate Oxidase	Produces H_2O_2 during reaction
1.2.3.5	Pyruvate Oxidase (Coa-Acetylating)	Produces H_2O_2 during reaction
1.2.3.6	Indole-3-Acetaldehyde Oxidase	Produces H_2O_2 during reaction
1.2.3.7	Pyridoxal Oxidase	Produces H_2O_2 during reaction
1.2.3.8	Aryl-Aldehyde Oxidase	Produces H_2O_2 during reaction
1.2.3.9	Reticuline Oxidase	Produces H_2O_2 during reaction

1.21.3.3	Tetrahydrocannabinolic Acid Synthase	Produces H_2O_2 during reaction
1.21.3.7	Cannabidiolic Acid Synthase	Produces H_2O_2 during reaction
1.21.3.8	Tryptophan Alpha,Beta-Oxidase	Produces H_2O_2 during reaction
1.3.3.10	Pyrroloquinoline-Quinone Synthase	Produces H_2O_2 during reaction
1.3.3.11	L-Galactonolactone Oxidase	Produces H_2O_2 during reaction
1.3.3.12	Albonoursin Synthase	Produces H_2O_2 during reaction
1.3.3.13	Aclacinomycin-A Oxidase	Produces H_2O_2 during reaction
1.3.3.14	Coproporphyrinogen III Oxidase	Produces H_2O_2 during reaction
1.3.3.15	Oxazoline Dehydrogenase	Produces H_2O_2 during reaction
1.3.3.16	Protoporphyrinogen Oxidase	Produces H_2O_2 during reaction
1.3.3.4	Acyl-Coa Oxidase	Produces H_2O_2 during reaction
1.3.3.6	Dihydrouracil Oxidase	Produces H_2O_2 during reaction
1.3.3.7	Tetrahydroberberine Oxidase	Produces H_2O_2 during reaction
1.3.3.8	(R)-6-Hydroxynicotine Oxidase	Produces H_2O_2 during reaction
1.3.5.6	L-Pipecolate Oxidase	Produces H_2O_2 during reaction
1.3.5.7	D-Aspartate Oxidase	Produces H_2O_2 during reaction
1.4.3.1	Putrescine Oxidase	Produces H_2O_2 during reaction
1.4.3.10	L-Glutamate Oxidase	Produces H_2O_2 during reaction
1.4.3.11	Cyclohexylamine Oxidase	Produces H_2O_2 during reaction
1.4.3.12	Protein-Lysine 6-Oxidase	Produces H_2O_2 during reaction
1.4.3.13	L-Lysine Oxidase	Produces H_2O_2 during reaction
1.4.3.14	D-Glutamate(D-Aspartate) Oxidase	Produces H_2O_2 during reaction
1.4.3.15	L-Aspartate Oxidase (D-Glutamate Oxidase)	Produces H_2O_2 during reaction
1.4.3.16	Glycine Oxidase	Produces H_2O_2 during reaction
1.4.3.19	L-Amino-Acid Oxidase	Produces H_2O_2 during reaction
1.4.3.2	L-Lysine 6-Oxidase (L-Amino Acid Oxidase)	Produces H_2O_2 during reaction
1.4.3.20	Primary-Amine Oxidase	Produces H_2O_2 during reaction
1.4.3.21	Diamine Oxidase	Produces H_2O_2 during reaction
1.4.3.23	7-Chloro-L-Tryptophan Oxidase	Produces H_2O_2 during reaction
1.4.3.23	Pseudooxynicotine Oxidase	Produces H_2O_2 during reaction
1.4.3.24	L-Arginine Oxidase	Produces H_2O_2 during reaction
1.4.3.25	Pre-Mycofactocin Synthase	Produces H_2O_2 during reaction
1.4.3.26	D-Amino-Acid Oxidase	Produces H_2O_2 during reaction
1.4.3.3	Monoamine Oxidase (D-Amino Acid Oxidase)	Produces H_2O_2 during reaction
1.4.3.4	Pyridoxal 5'-Phosphate Synthase	Produces H_2O_2 during reaction
1.4.3.5	D-Glutamate Oxidase	Produces H_2O_2 during reaction
1.4.3.7	Ethanolamine Oxidase	Produces H_2O_2 during reaction
1.4.3.8	Sarcosine Oxidasee	Produces H_2O_2 during reaction
1.5.3.1	Dimethylglycine Oxidase	Produces H_2O_2 during reaction
1.5.3.10	Dihydrobenzophenanthridine Oxidase	Produces H_2O_2 during reaction

1.5.3.12	N(1)-Acetylpolyamine Oxidase	Produces H_2O_2 during reaction
1.5.3.13	Polyamine Oxidase	Produces H_2O_2 during reaction
1.5.3.14	N(8)-Acetylspermidine Oxidase	Produces H_2O_2 during reaction
1.5.3.15	Spermine Oxidase	Produces H_2O_2 during reaction
1.5.3.16	Non-Specific Polyamine Oxidase	Produces H_2O_2 during reaction
1.5.3.17	L-Saccharopine Oxidase	Produces H_2O_2 during reaction
1.5.3.18	4-Methylaminobutanoate Oxidase	Produces H_2O_2 during reaction
1.5.3.19	N-Methyl-L-Amino-Acid Oxidase	Produces H_2O_2 during reaction
1.5.3.2	N-Alkylglycine Oxidase (Amino Acid Oxidase)	Produces H_2O_2 during reaction
1.5.3.20	4-Methylaminobutanoate Oxidase	Produces H_2O_2 during reaction
1.5.3.21	Sarcosine Oxidase	Produces H_2O_2 during reaction
1.5.3.24	N(6)-Methyl-Lysine Oxidase	Produces H_2O_2 during reaction
1.5.3.4	(S)-6-Hydroxynicotine Oxidase	Produces H_2O_2 during reaction
1.5.3.5	NAD(P)H Oxidase	Produces H_2O_2 during reaction
1.6.3.1	NADH Oxidase	Produces H_2O_2 during reaction
1.6.3.3	NADH Oxidase	Produces H_2O_2 during reaction
1.6.3.5	Renalase	Produces H_2O_2 during reaction
1.7.3.1	Nitroalkane Oxidase	Produces H_2O_2 during reaction
1.7.3.3	Factor Independent Urate Hydroxylase	Produces H_2O_2 during reaction
1.7.3.5	3-Aci-Nitropropanoate Oxidase	Produces H_2O_2 during reaction
1.8.3.1	Sulfite Oxidase	Produces H_2O_2 during reaction
1.8.3.2	Thiol Oxidase	Produces H_2O_2 during reaction
1.8.3.3	Glutathione Oxidase	Produces H_2O_2 during reaction
1.8.3.4	Methanethiol Oxidase	Produces H_2O_2 during reaction
1.8.3.5	Prenylcysteine Oxidase	Produces H_2O_2 during reaction
1.8.3.6	Farnesylcysteine Lyase	Produces H_2O_2 during reaction
1.1.99.18	Cellobiose Dehydrogenase	Produces H_2O_2 during reaction
1.1.99.1	Choline Dehydrogenase	Produces H_2O_2 during reaction

Curated list of enzymes with putative roles in direct or indirect Mn(II) oxidation. Enzymes were selected based on known roles in Mn cycling or production of reactive oxygen species known to oxidize Mn. This curated list was used for correlation analysis as the suite of putative Mn-oxidizing enzymes.

Supplementary Table 5. Linear correlation statistics between relative abundance of different analyzed FT-ICR MS functional groups and the relative expression of CAZy classified enzyme groups

	Carbohydrate Esterases (CE)		*Glycoside Hydrolases (GH)*		*Glycosyltransferases (GT)*		*Polysaccharide Lyases (PL)*		*Auxiliary Activity Enzymes (AA)*	
Functional Groups	R^2	*p-value*	R^2	*p-value*	R^2	*p-value*	R^2	*p-value*	R^2	*p-value*
Amino Sugars	0.029	0.33	0.0041	0.71	0.036	0.27	0.014	0.48	0.017	0.46
Carbohydrates	0.04	0.26	0.029	0.33	**0.18**	**0.01***	0.0046	0.7	0.084	0.10
Condensed Hydrocarbons	0.017	0.46	0.017	0.47	0.044	0.23	0.014	0.48	0.32	0.29
Lignin	**0.15**	**0.02***	**0.19**	**0.009***	**0.18**	**0.01***	0.05	0.20	**0.14**	**0.02***
Lipid	0.044	0.23	0.073	0.11	0.078	0.098	0.063	0.14	0.078	0.10
Protein	0.0004	0.97	0.0058	0.66	0.032	0.30	0.0027	0.76	0.063	0.14
Tannin	0.048	0.19	0.058	0.16	0.014	0.50	0.048	0.21	0.012	0.54
Unsaturated Hydrocarbons	0.0081	0.61	0.0002	0.93	0.01	0.56	0.032	0.29	0.017	0.46

Linear regression adjusted R^2 and p-values of the fitted correlations between the relative gene expression of CAZy classified enzymes (normalized to total mRNA) and the relative abundance of different functional groups analyzed using FT-ICR MS analysis.
Correlations bolded with (*) notation indicates statistically significant correlations ($p < 0.05$) for samples (n=4). Significance was determined using the F statistic for the drawn linear correlation.

Supplementary Table 6. Sequential extraction and XANES fitting results

a) Day 7

	Extractions				*XANES fitting*		
	Water (µg/g)	*Ammonium Acetate (µg/g)*	*Sodium Pyrophosphate(µg/g)*	*Dithionite – HCl (µg/g)*	*Mn(II)*	*Mn(III)*	*Mn(IV)*
Control							
Oxic	6.05 ± 1.09	3.34 ± 0.31	11.37 ± 1.52	4.34 ± 0.26	0.358	0.39	0.252
OAI	5.39 ± 0.91	2.11 ± 0.42	8.55 ± 1.20	4.92 ± 0.72	0.542	0.290	0.168
Anoxic	4.82 ± 0.52	2.35 ± 0.57	7.34 ± 1.57	3.90 ± 0.28	0.129	0.332	0.540
Low Mn							
Oxic	9.49 ± 1.75	5.64 ± 1.28	12.88 ± 4.15	4.92 ± 0.91	0.304	0.326	0.369
OAI	11.68 ± 2.33	8.58 ± 1.83	17.02 ± 5.63	5.13 ± .095	0.308	0.323	0.370
Anoxic	19.29 ± 2.68	14.65 ± 1.77	24.50 ± 3.39	8.05 ± 1.49	0.178	0.362	0.460
High Mn							
Oxic	6.50 ± 0.90	2.72 ± 0.34	8.10 ± 3.10	5.46 ± 1.23	0.337	0.001	0.664
OAI	9.08 ± 1.10	7.28 ± 1.48	16.18 ± 4.22	5.06 ± 0.88	0.266	0.341	0.393
Anoxic	28.46 ± 9.10	44.95 ± 17.14	70.65 ± 30.66	13.50 ± 3.83	0.546	0.178	0.276

b) Day 30

	Extractions				*XANES fitting*		
	Water (µg/g)	*Ammonium Acetate (µg/g)*	*Sodium Pyrophosphate(µg/g)*	*Dithionite – HCl (µg/g)*	*Mn(II)*	*Mn(III)*	*Mn(IV)*
Control							
Oxic	4.81 ± 0.74	1.58 ± 0.28	10.62 ± 2.85	10.15 ± 3.84	0.237	0.295	0.469
OAI	3.89 ± 1.14	1.51 ± 0.47	5.31 ± 0.62	12.66 ± 8.30	0.214	0.277	0.509
Anoxic	3.73 ± 0.77	1.48 ± 0.32	9.27 ± 2.59	4.56 ± 0.24	0.229	0.244	0.527
Low Mn							
Oxic	39.11 ± 2.62	23.77 ± 9.08	39.91 ± 22.18	10.34 ± 2.26	0.535	0.184	0.282
OAI	30.76 ± 4.93	40.42 ± 11.44	69.72 ± 21.08	14.55 ± 2.53	0.343	0.234	0.422
Anoxic	25.05 ± 1.84	15.85 ± 2.17	22.45 ± 3.04	10.76 ± 0.68	0.285	0.218	0.498
High Mn							
Oxic	33.93 ± 7.77	185.49 ± 74.83	333.80 ± 138.12	45.13 ± 16.77	0.562	0.198	0.240
OAI	42.60 ± 6.12	294.76 ± 105.43	1373.03 ± 595.86	117.52 ± 54.14	0.494	0.256	0.249
Anoxic	69.77 ± 10.70	213.61 ± 66.78	308.23 ± 33.29	52.08 ± 5.54	0.652	0.182	0.166

c) Day 90

	Extractions				XANES fitting		
	Water (μg/g)	*Ammonium Acetate (μg/g)*	*Sodium Pyrophosphate(μg/g)*	*Dithionite – HCl (μg/g)*	*Mn(II)*	*Mn(III)*	*Mn(IV)*
Control							
Oxic	7.67 ± 2.24	2.30 ± 0.45	6.55 ± 0.49	3.49 ± 0.22	0.310	0.240	0.450
OAI	2.13 ± 0.84	2.18 ± 0.63	6.64 ± 1.36	3.42 ± 0.37	0.300	0.245	0.455
Anoxic	3.47 ± 1.05	2.17 ± 0.45	5.53 ± 0.85	3.45 ± 0.08	0.335	0.230	0.435
Low Mn							
Oxic	55.03 ± 25.96	43.42 ± 8.60	113.27 ± 33.80	42.97 ± 24.62	0.615	0.137	0.248
OAI	14.31 ± 3.23	27.55 ± 5.22	53.74 ± 13.44	14.19 ± 2.78	0.556	0.153	0.290
Anoxic	13.39 ± 2.29	22.22 ± 8.14	46.19 ± 20.34	14.39 ± 3.20	0.651	0.134	0.216
High Mn							
Oxic	87.08 ± 28.92	215.84 ± 70.60	578.30 ± 196.16	73.02 ± 24.91	0.670	0.187	0.143
OAI	27.15 ± 6.47	154.46 ± 32.56	581.07 ± 159.13	69.00 ± 23.81	0.646	0.242	0.112
Anoxic	39.48 ± 5.62	225.79 ± 26.57	633.53 ± 155.25	100.10 ± 10.90	0.659	0.289	0.052

Sequential extraction, ordered in sequence from water, ammonium acetate, sodium pyrophosphate, and dithionite-HCl and XANES fittings for solid-phase samples. Sequential extraction results are averages of each extractant with standard error (n=4). Samples were run on an ICP-MS. XANES fitting was done in Athena and utilized to determine total Mn(II), Mn(III), and Mn(IV) using the sum of the sequential extractions for reactor samples.

a)

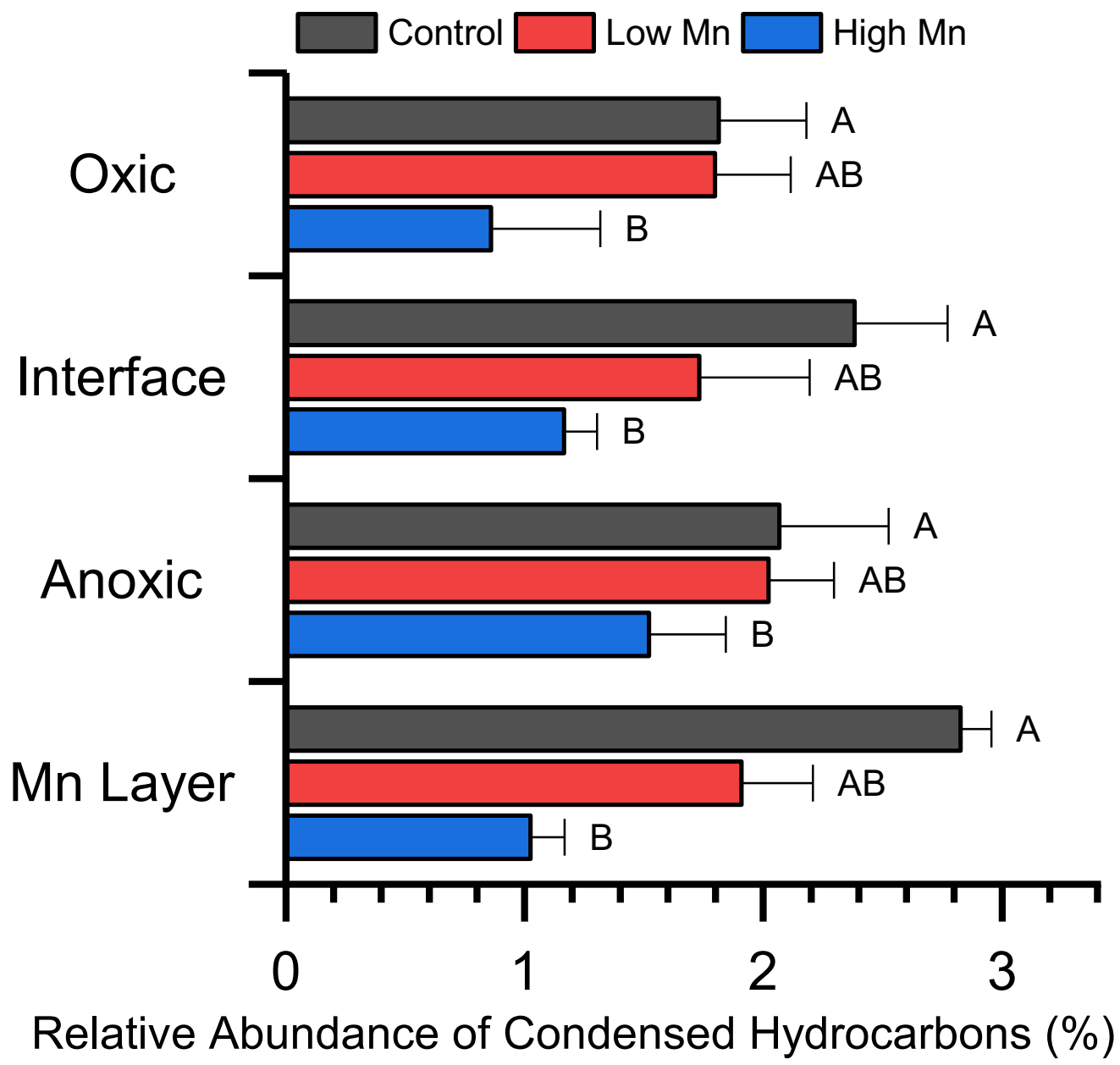

b)

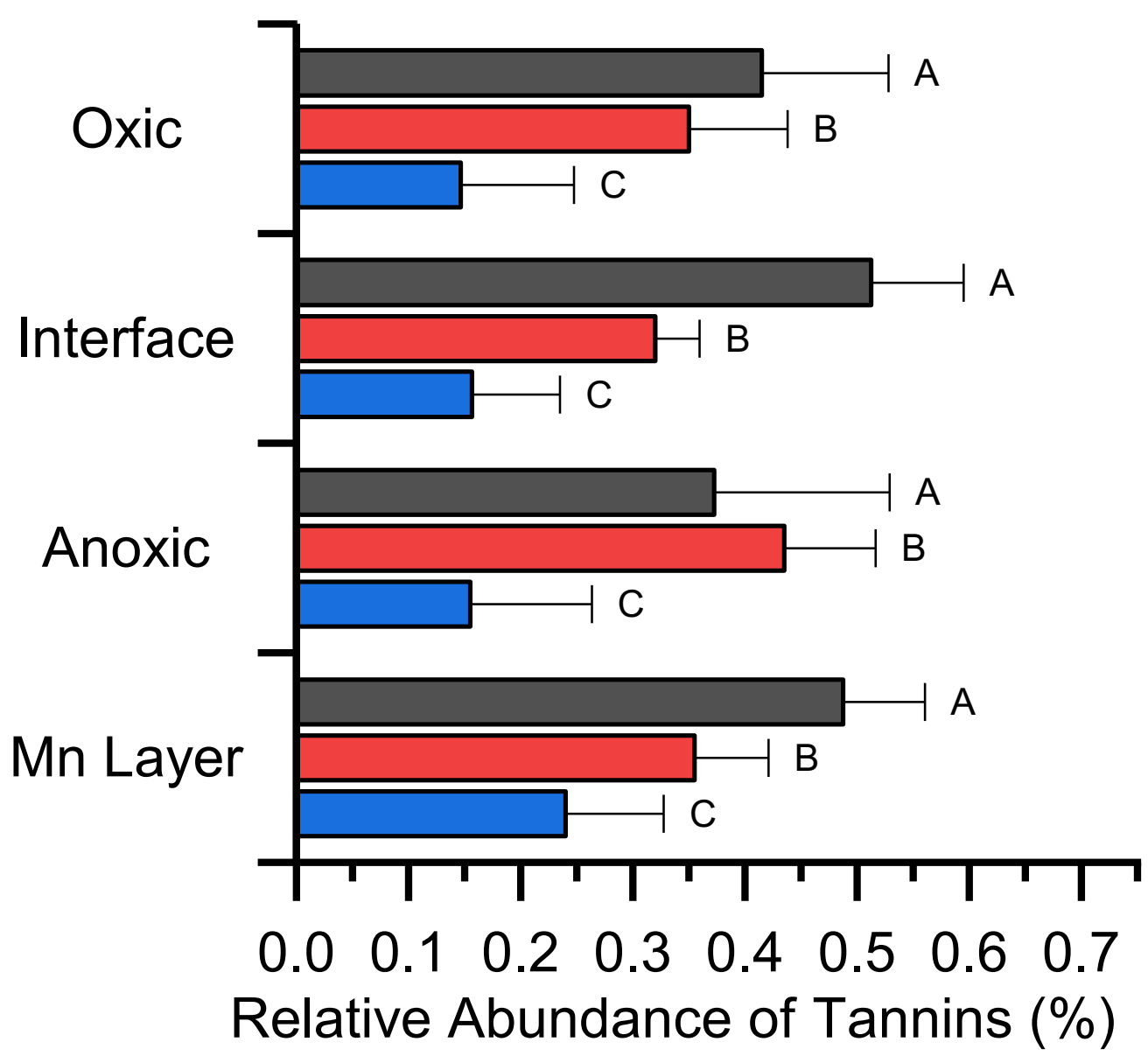

Supplementary Figure 1: Relative abundance of condensed hydrocarbons and tannins across the oxic-anoxic interface at day 30. *FT-ICR MS analysis on water-methanol extractions of reactor samples across the oxic-anoxic interface. The relative abundance of two different aromatic compounds are shown in a) condensed hydrocarbon compounds and b) tannin compounds (n=4). Statistical analysis was conducted within each sampling site of the reactors, comparing the effect of different treatments using a one-way ANOVA Tukey test. Letters denote statistically significant treatments within each sampling zone ($p < 0.05$).*

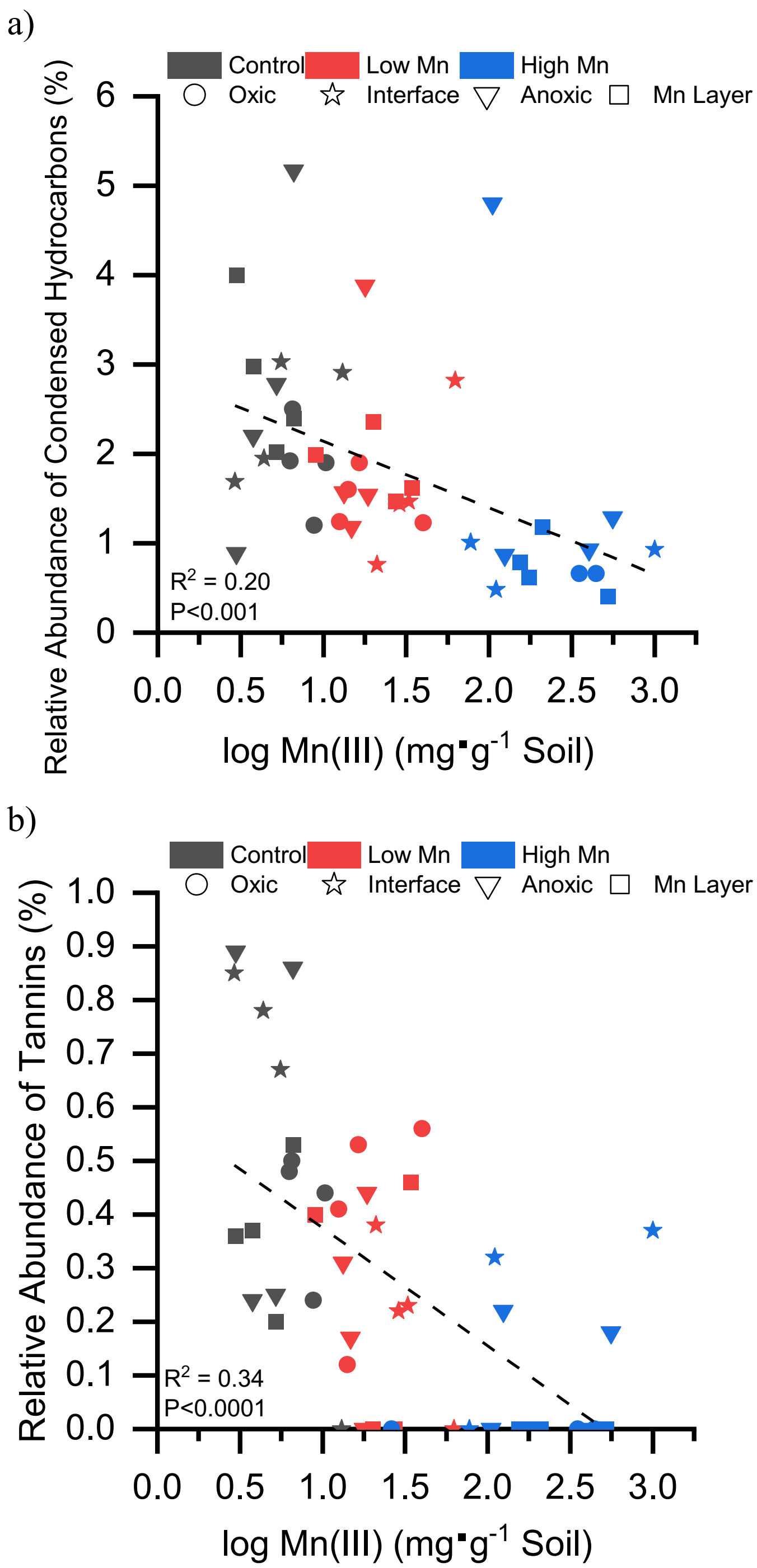

Supplementary Figure 2: Log-transformed Mn(III) concentrations compared to the relative abundance of condensed hydrocarbons and tannins *Averages (n=4) of the relative abundance of a) condensed hydrocarbons and b) tannins analyzed using FT-ICR MS on methanol-water extractions compared to log-transformed Mn(III) concentrations determined through Mn XANES and sequential extractions. Linear regressions were drawn to fit data, with the adjusted* R^2 *and significance (*$p < 0.05$*) determined using the F-statistic for the drawn correlation*

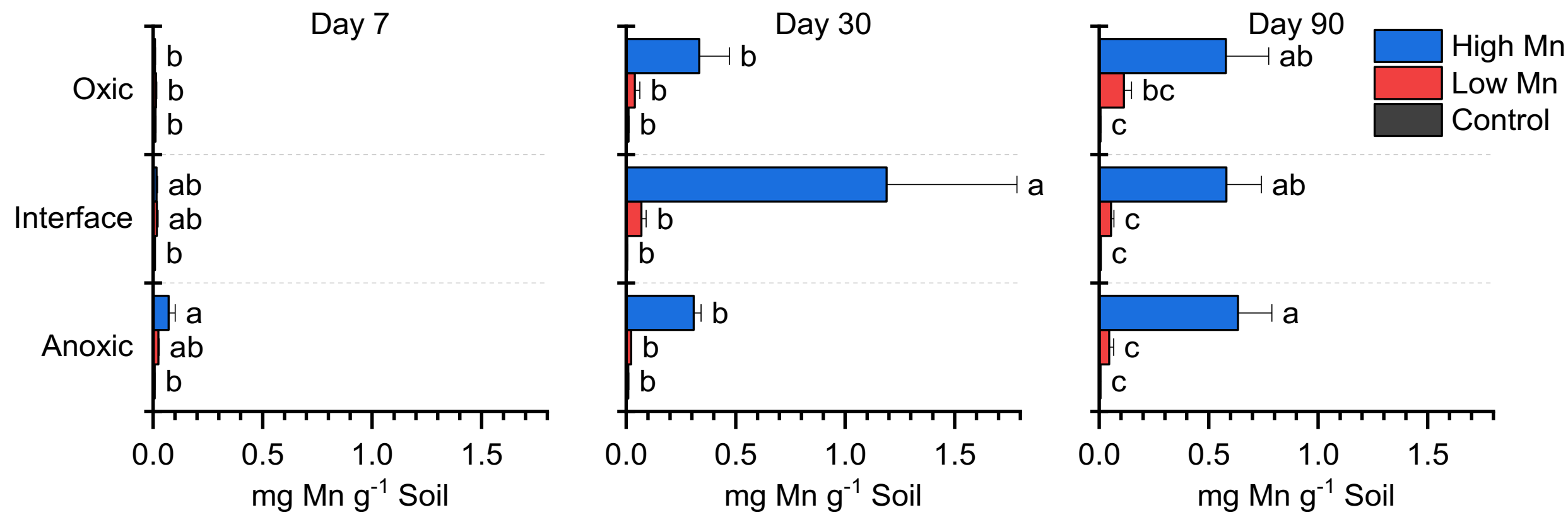

Supplementary Figure 3: Pyrophosphate Extractable Mn at days 7, 30, and 90. *Mn concentrations as determined by sodium-pyrophosphate extractions. Statistical analysis was done using a 2-way ANOVA using treatment and sampling location, with error bars denoting standard error (n=4). Lettering denotes statistical significance within each sampling timepoint to other treatments across the oxic-anoxic interface ($p < 0.05$).*

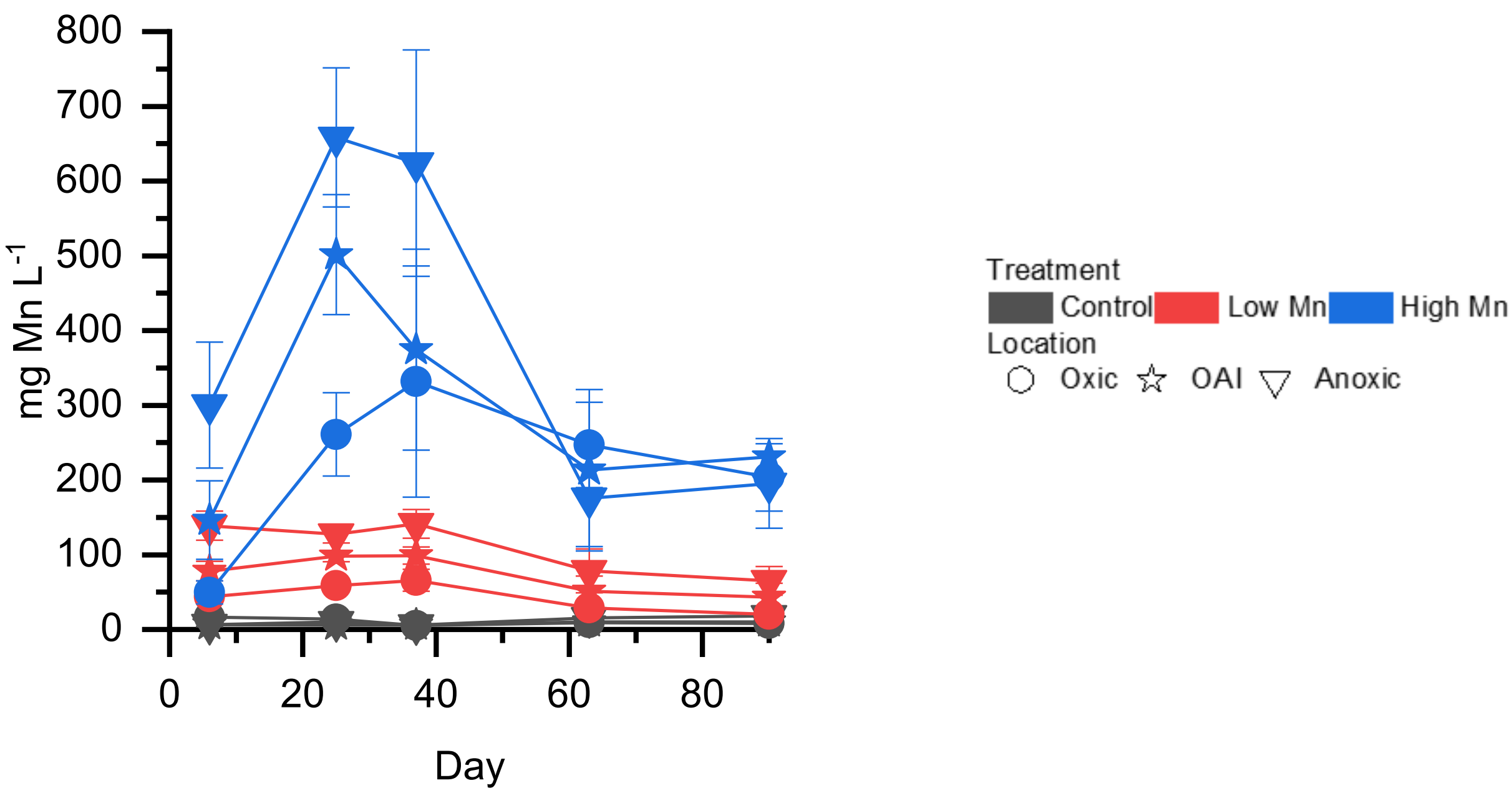

Supplementary Figure 4: Concntration of porewater extractable Mn. *Pore water measurements of total Mn taken from MicroRhizon samplers installed at each sampling zone conducted throughout the experiment analyzed using an ICP-MS. Error bars represent standard error for each porewater collection period (n=4).*

a)

b)

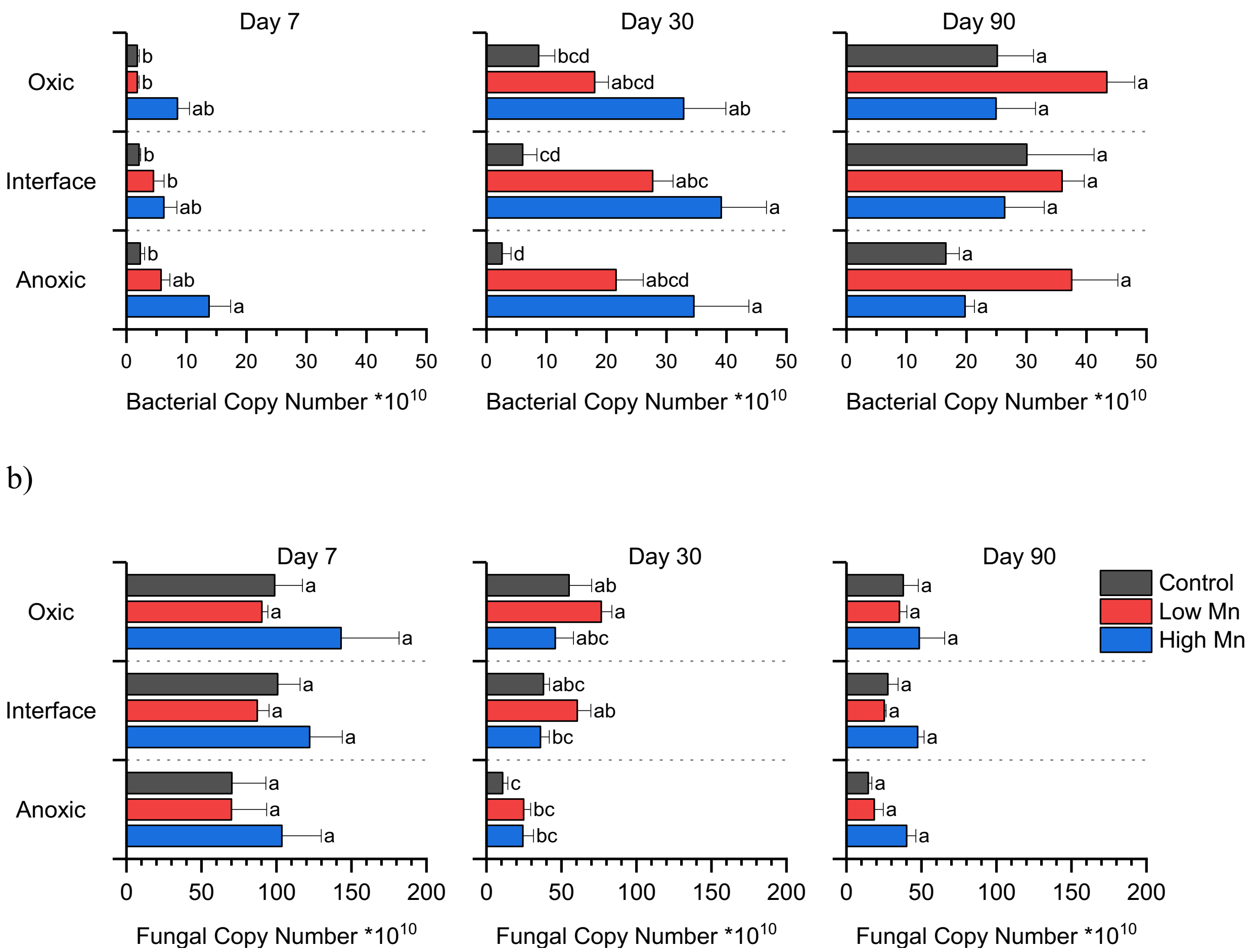

Supplementary Figure 5: Bacterial and fungal abundance at days 7, 30, and 90 *qPCR results for relative (a) bacterial and (b) fungal abundance conducted on DNA extractions of reactor samples. Error bars denote the standard error, while letters indicate statistical significance among groupings (n=4, p < 0.05). Statistical comparisons were done using a 3-way ANOVA to compare between treatment, sampling location, and timepoint.*

a)

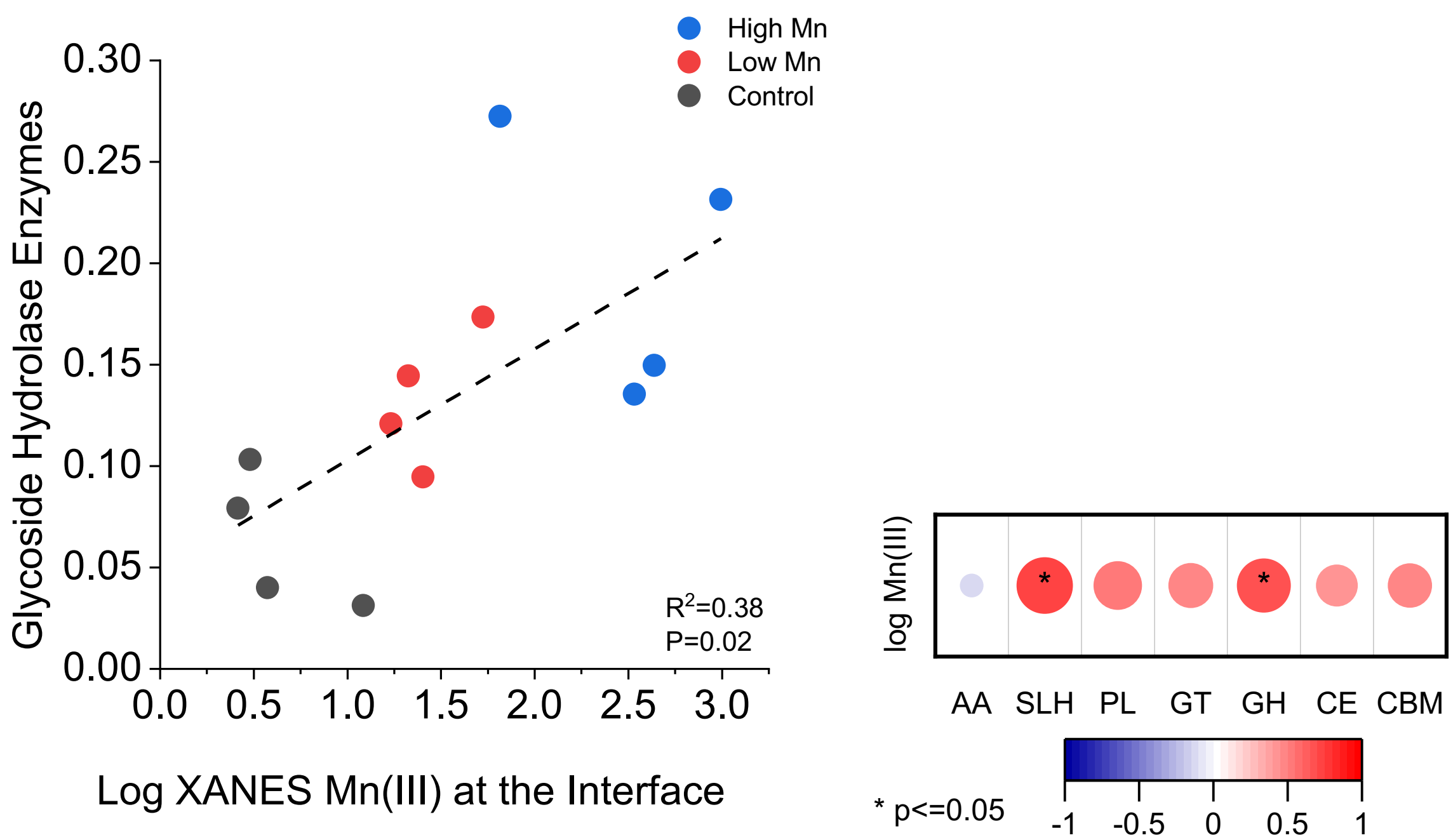
High Mn
Low Mn
Control
Glycoside Hydrolase Enzymes
0.30
0.25
0.20
0.15
0.10
0.05
0.00
0.0
0.5
1.0
1.5
2.0
2.5
3.0
Log XANES Mn(III) at the Interface
R^2=0.38
P=0.02
log Mn(III)
AA
SLH
PL
GT
GH
CE
CBM
*
* p<=0.05
-1
-0.5
0
0.5
1

b)

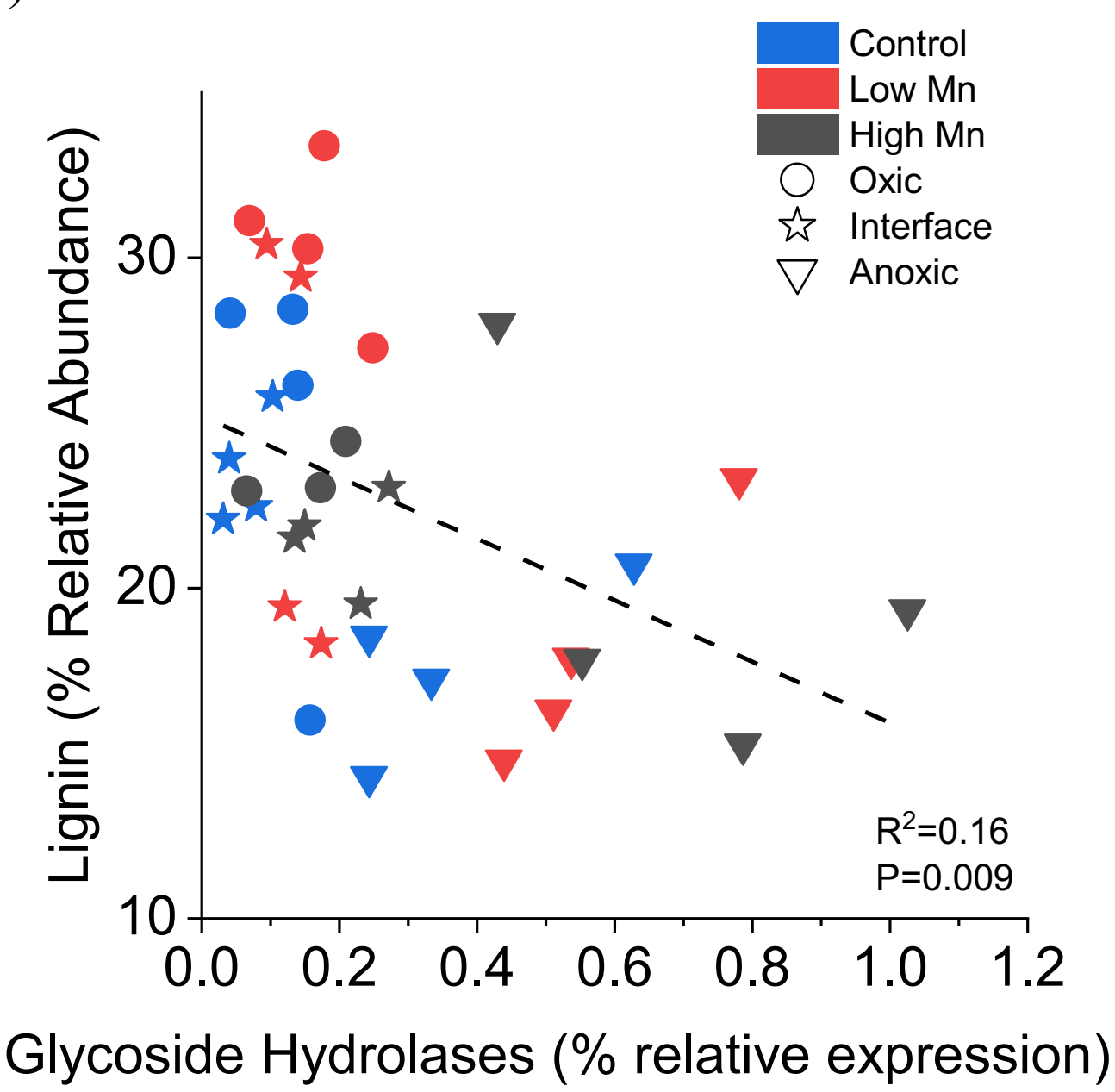
Control
Low Mn
High Mn
Oxic
Interface
Anoxic
Lignin (% Relative Abundance)
30
20
10
0.0
0.2
0.4
0.6
0.8
1.0
1.2
Glycoside Hydrolases (% relative expression)
R^2=0.16
P=0.009

c)

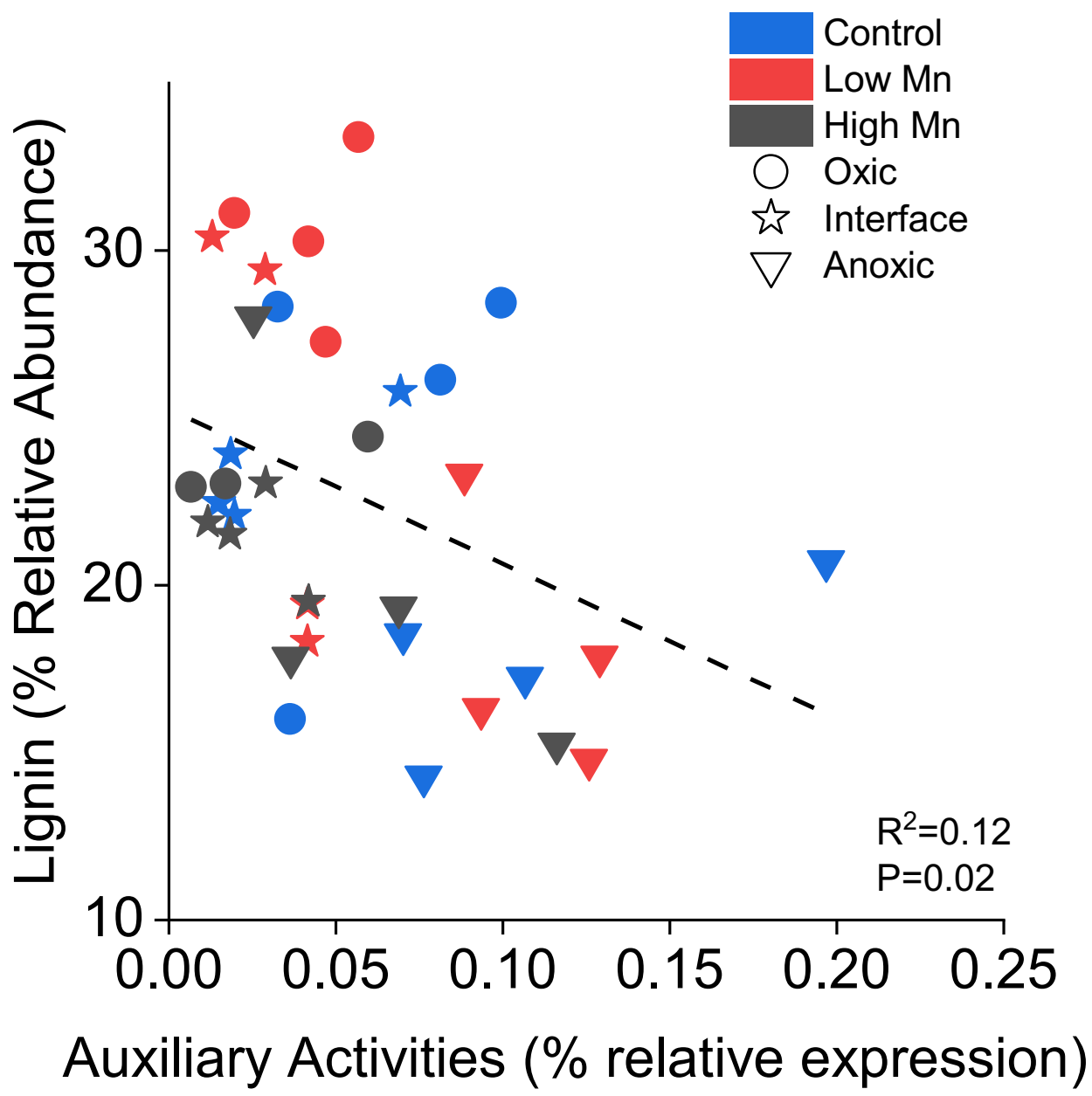

Supplementary Figure 6: Correlations between CAZy gene expression to Mn(III) formation at the oxic-anoxic interface and lignin abundance *Expression of CAZy enzyme expression through metatranscriptomics analysis to measurements of Mn(III) and relative abundance of lignin. Specifically, we a) compared glycoside hydrolases and all other CAZy enzyme categories (inset) to log-transformed Mn(III) formation at OAIs. We then compared the relative abundance of lignin in FT-ICR MS analysis to the relative expression of both b) glycoside hydrolases (GH) and c) auxiliary activity (AA) enzymes. A linear regression was established for CAZy – FT-ICR MS comparisons and significance was determined based on an F-test. A comprehensive table covering all analyzed relationships can be found in Table S5.*

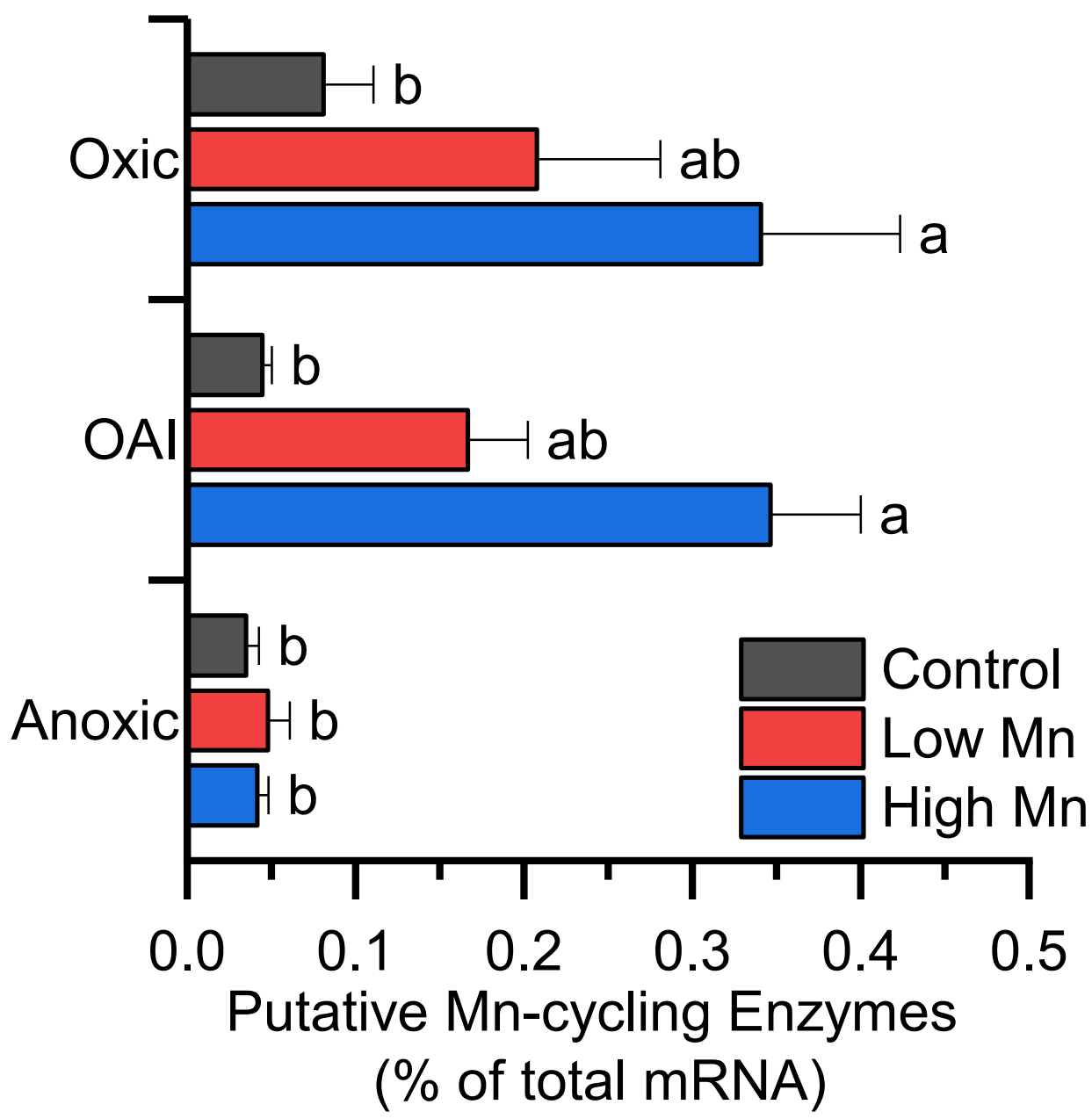

Supplementary Figure 7. Relative expression of putative-Mn cycling enzymes across sampling zones and treatments *Specific list for Mn-cycling enzymes can be found in Table S-4. Error bars denote the standard error. Statistical comparisons were done using a 2-way ANOVA to compare between treatment and sampling location, wherein groups that do not share any letters are statistically different (n=4, p < 0.05).*

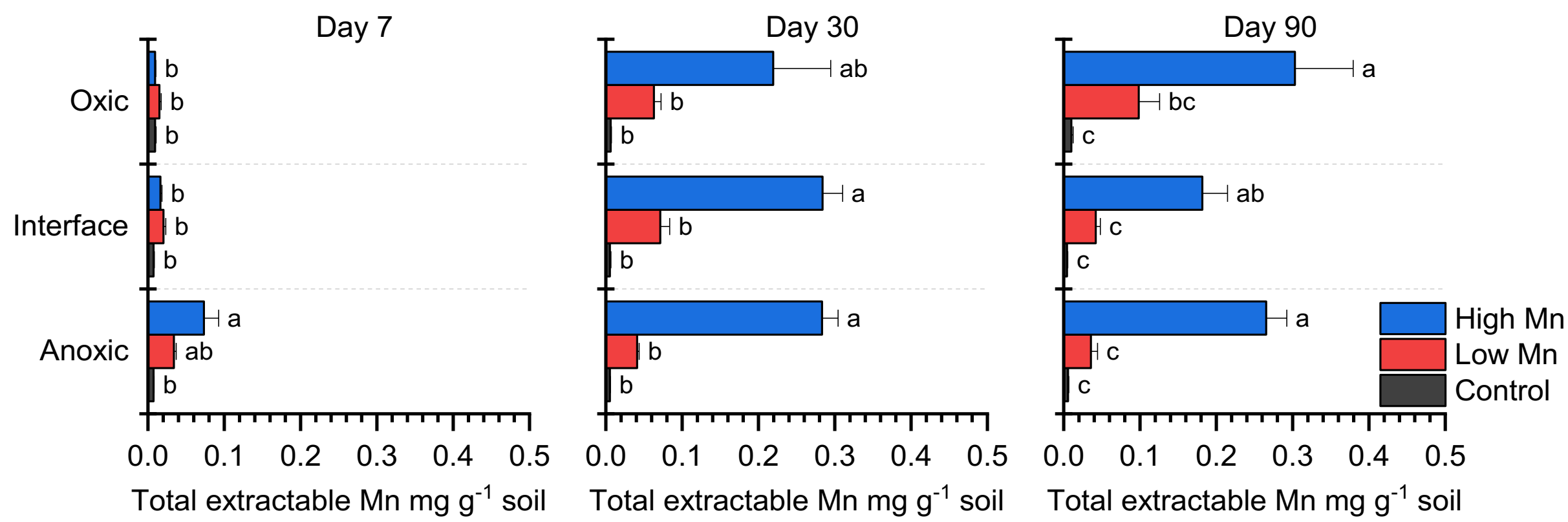

Supplementary Figure 8. Combined water and ammonium acetate extractable Mn at days 7, 30, and 90 *Quantitative measurements of Mn using sequential extractions. Here we combined both water extractable (available Mn pool) and ammonium acetate extractable (exchangeable Mn pool) together and compared the extractions within each timepoint using a 2-way ANOVA test. Error bars denote the standard error while lettering represents statistical significance between treatments and across the oxic-anoxic interface within each timepoint (n=4, $p < 0.05$).*

a)
b)

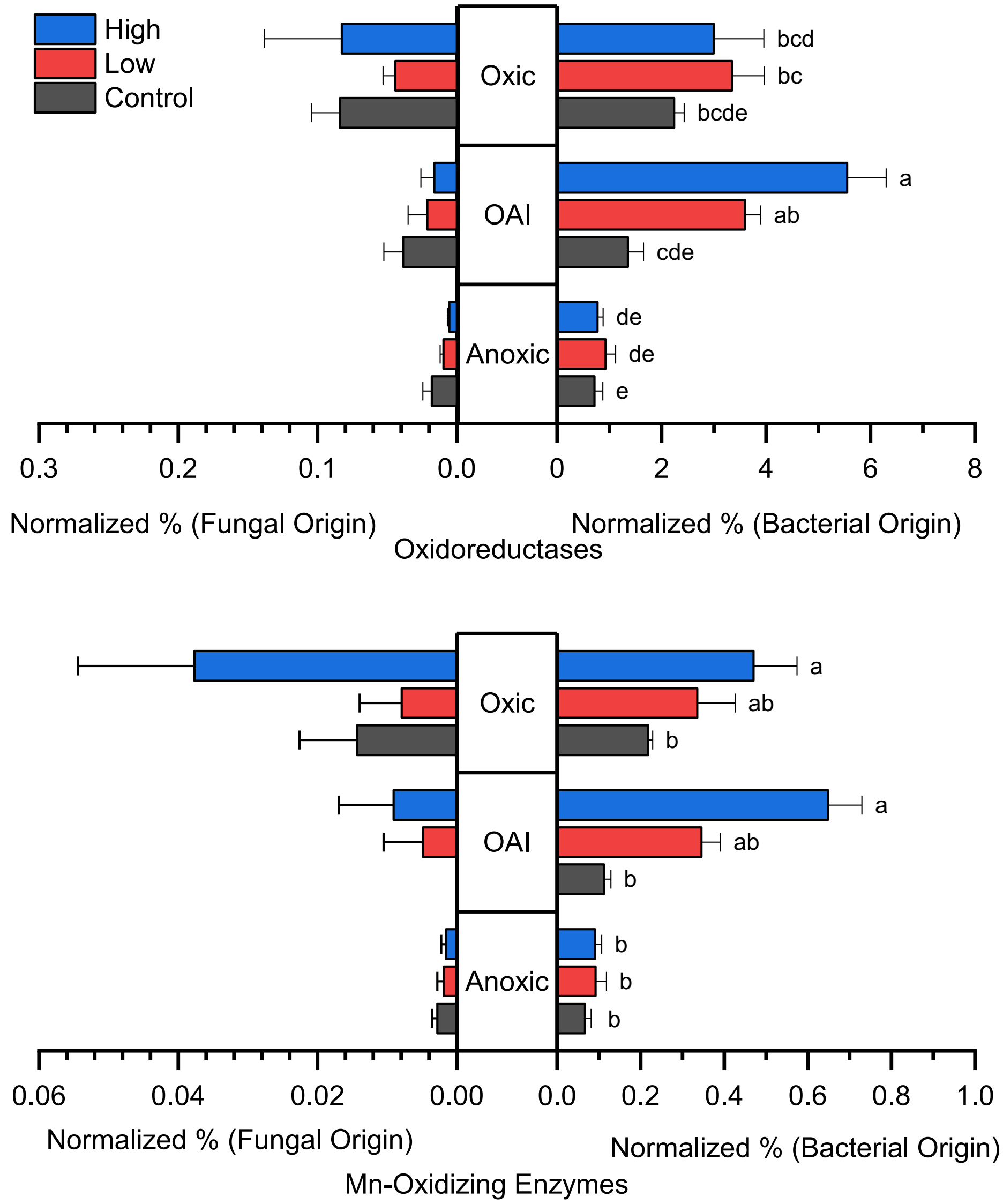

Supplementary Figure 10. Relative expression of oxidoreductases and putative Mn(II)-oxidizing enzymes between fungi and bacteria Relative expression of both *a) oxidoreductase and b) putative Mn-oxidizing enzyme gene expression across the oxic-anoxic interface parsed by fungal (left x-axis) and bacterial (right x-axis) origin. Statistical comparisons were done using a 2-way ANOVA to compare between treatment and sampling location. Distinct letters represent significantly different groupings (n=4, $p < 0.05$). Lettering was not established for fungi because they were not statistically significant.*

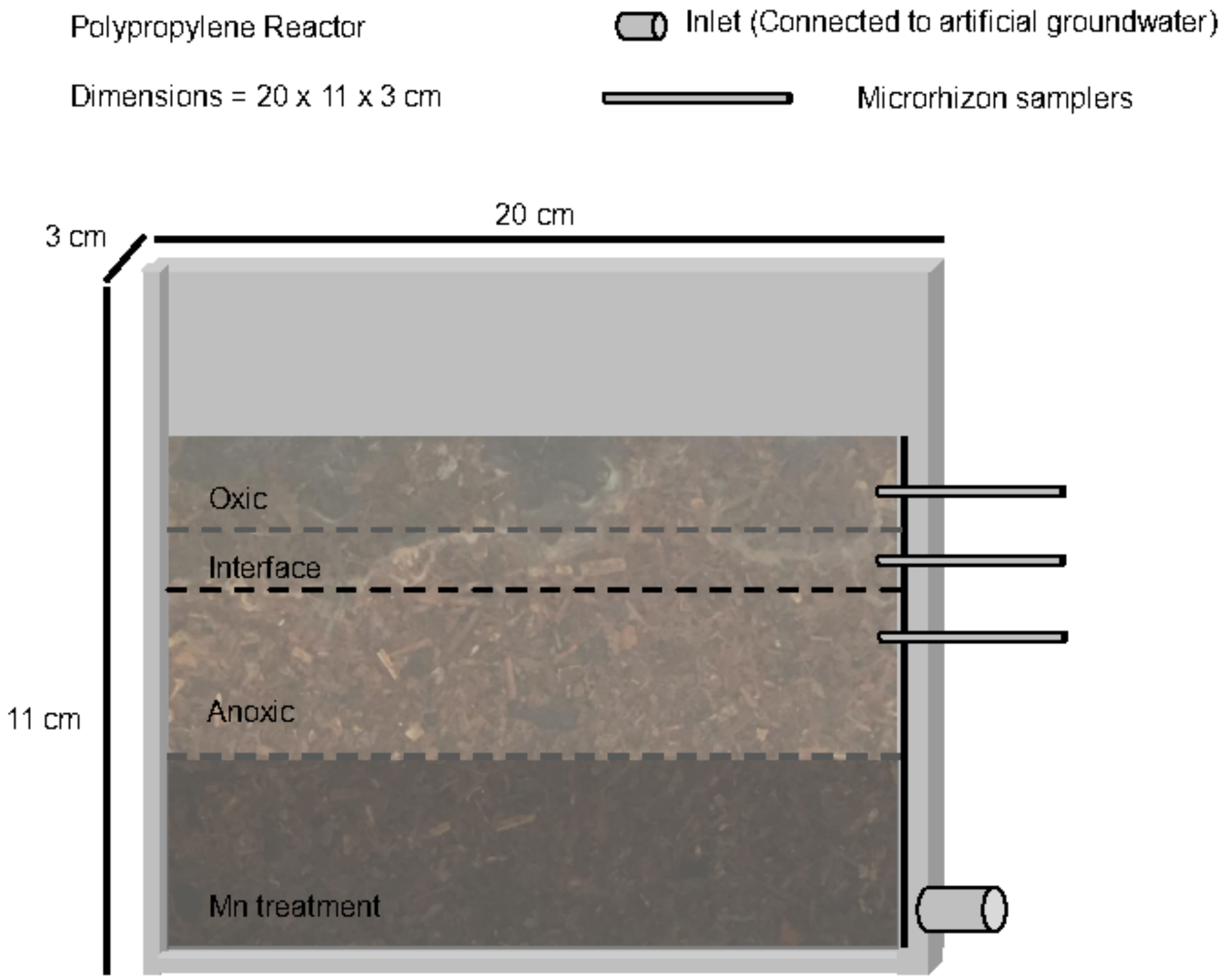

Supplementary Figure 11. Schematic of diffusion reactors Diffusion reactors were established using a polypropylene shell (20 x 11 x 3 cm). The total soil column height within the reactors was 75 mm. The oxic-anoxic interface was established by connecting the reactor to a source of artificial groundwater using an inlet at the bottom of the reactor and maintaining the water table height at 15 mm. We designated the top 0-10 mm below the soil surface as the oxic zone, 10-15 mm as the oxic-anoxic interface, and 15-50 mm as the anoxic zone. Microrhizon samplers were established at each sampling zone for porewater sampling. Depths 50-75 mm below the surface marked the bottom layer receiving Mn(III/IV) additions.
