# Supplementary material for: Synergy between reactive manganese species and enzymes drives litter decomposition hotspots
Source: Nat Commun. 2026 Jul 25;17:10355. doi: 10.1038/s41467-026-75920-x (PMC13623943; doi:10.1038/s41467-026-75920-x)
Supplement: Supplementary file 3 [file 41467_2026_75920_MOESM3_ESM.pdf]

nature portfolio

Peer Review File

Synergy between reactive manganese species and enzymes drives litter decomposition hotspots

Corresponding Author: Professor Marco Keiluweit

This file contains all reviewer reports in order by version, followed by all author rebuttals in order by version.

Version 0:

Reviewer comments:

Reviewer #1

(Remarks to the Author)
NCOMMS-25-100259
Synergy between reactive metals and enzymes drives hotspots of litter decomposition

This is a well described, clearly evidenced and elegantly designed experimental study into the transient and spatially-constrained role of redox interfaces as hotspots and hot moments, within the complex and dynamic soil system.

The background is well framed and scientific problem and its significance is clearly stated. The experimental approach neatly addresses the key research questions integrating a range of techniques. Figures are well produced, effectively illustrate and record the key results, and the captions are clear and detailed. The methodology appears sound and is thoroughly described, with the minor exception of the points noted below. I did not find any significant flaws or questionable points within the data presented and the method is described in sufficient detail to enable replication.

The 'activation' of decomposition by Mn oxidation at the oxic-anoxic interface allowing attack of litter by a wide spectrum of microbial pathways, rather than the specialist oxidative depolymerization enzymes, is an important finding which should drive a significant new parameterisation of soil biogeochemical models.

The specific experimental setup used here created a distinct 'hot moment' which the authors determine is limited by Mn(II) oxidation within the oxic zone that regulates supply of Mn to the oxic-anoxic interface. In the experiments this takes c. 30 days to develop to peak activity, a timescale which in dynamic soil environments may accommodate several cycles of wetting-drying and hence shorter-lived oxic-anoxic interfaces. While the 'hotspot' element of the findings remains an interesting and important insight, the implications of this 'hot moment' are less well explored in the discussion either as a limitation of the experimental design, a consideration for modelling this phenomenon, or as a potential key focus for future research.

Specific points:

Line 340 doesn't make good sense - presumably the design is modified from reactors used by ref 53, but as written this doesn't scan correctly. What was the modification from the original design and why? (lines 341-348)

Line 377-378 - sentence introducing and describing the artificial soil solution appears incomplete / concatenated / duplicated.

Reviewer #2

(Remarks to the Author)
The present manuscript addresses an interesting and relevant topic concerning the role of manganese (Mn) availability in regulating litter decomposition across oxic-anoxic interfaces in terrestrial ecosystems. The authors designed efficient soil reactors to systematically investigate the hotspots of litter decomposition, with a specific focus on the interplay between

reactive Mn species and microbial enzyme activity. They reported that stimulating microbial production of reactive manganese(III) at the oxic–anoxic interfaces simultaneously doubled the expression levels of diverse litter-decomposing enzymes, which in turn significantly accelerated the rate of litter decomposition. This is a novel and valuable finding that can substantially improve our current understanding of the complex mechanisms underlying litter decomposition, a key process controlling carbon cycling in terrestrial environments.
Generally speaking, the manuscript is well-prepared, with a logical structure and a well-designed experimental framework that effectively tests the research hypotheses. However, I have one major concern regarding the litter material used in the study, the exclusive use of broadleaf litter, without the inclusion of needle litter. Given that needle litter differs significantly from broadleaf litter in terms of chemical composition (e.g., lignin content) and decomposition dynamics, this exclusion may limit the generalizability of the study's findings. Below are some specific comments and suggestions.

Using "Mn" or "Manganese" would be more precise than "metals." The current title lacks specificity, as it does not explicitly highlight the central role of Mn, which is the core focus of the study.
The abstract is overly concise and lacks key experimental data. I suggest supplementing it with specific quantitative results to better showcase the study's contributions and enhance the abstract's informativeness.

Lines 26–28, the claim that "the assumption that oxidative enzymatic decomposition is maximized under fully oxic conditions" is widely accepted does not align with existing literature. In my opinion, numerous studies have demonstrated that many enzymes can maintain or even enhance their activity in partially anoxic environments, such as under snow cover or frozen conditions. For instance, several studies have reported that freeze-thaw cycles can promote enzyme activity by breaking down litter structure and increasing substrate accessibility.

The novelty of the study lies primarily in the role of Mn availability at oxic–anoxic interfaces, rather than in the regulation of enzyme activity during litter decomposition itself. Therefore, I suggest that the authors directly introduce Mn and its role in the first paragraph of the introduction, rather than focusing solely on oxidative decomposition, to better highlight the study's unique contribution.

Line 100, the diffusion reactors and other methods used to identify decomposition hotspots should be described in earlier paragraphs.

Lines 109–121 currently appear in an inappropriate section; this content should be included in the Methods section, as it describes experimental procedures. The Results section should focus on presenting key observations and findings, rather than methodological details.

The subsection starting at Line 160 is excessively long and difficult to follow. I recommend streamlining this section to emphasize the most important observations, rather than detailing every result. For example, the last two paragraphs of this subsection could be moved to a separate subsection to improve readability.

The limitations associated with the litter type used (i.e., the exclusion of needle litter) should be thoroughly discussed in the Discussion section, including how this may affect the interpretation and generalizability of the results.

Lines 351–353, the authors should clearly explain the rationale for selecting the specific litter types used in the study. Detailed information on litter quality and Mn concentrations should be provided.

Finally, the reactor design should be briefly described with a clear diagram. A visual illustration would help readers quickly understand the experimental setup.

Version 1:

Reviewer comments:

Reviewer #1

(Remarks to the Author)
I have reviewed the revisions against the comments I made on the original manuscript. I am satisfied that my specific concerns related to the description of the modified reactor and the soil solution have been addressed. Recommend accept for publication.

Reviewer #2

(Remarks to the Author)
no further comments

Open Access This Peer Review File is licensed under a Creative Commons Attribution 4.0 International License, which permits use, sharing, adaptation, distribution and reproduction in any medium or format, as long as you give appropriate credit to the original author(s) and the source, provide a link to the Creative Commons license, and indicate if changes were made.
In cases where reviewers are anonymous, credit should be given to 'Anonymous Referee' and the source.
The images or other third party material in this Peer Review File are included in the article's Creative Commons license, unless indicated otherwise in a credit line to the material. If material is not included in the article's Creative Commons license and your intended use is not permitted by statutory regulation or exceeds the permitted use, you will need to obtain permission directly from the copyright holder.
To view a copy of this license, visit https://creativecommons.org/licenses/by/4.0/

Response to reviewer comments: 2026-05-11

REVIEWER COMMENTS

Reviewer #1 (Remarks to the Author):

NCOMMS-25-100259
Synergy between reactive metals and enzymes drives hotspots of litter decomposition

This is a well described, clearly evidenced and elegantly designed experimental study into the transient and spatially-constrained role of redox interfaces as hotspots and hot moments, within the complex and dynamic soil system.

The background is well framed and scientific problem and its significance is clearly stated. The experimental approach neatly addresses the key research questions integrating a range of techniques. Figures are well produced, effectively illustrate and record the key results, and the captions are clear and detailed. The methodology appears sound and is thoroughly described, with the minor exception of the points noted below. I did not find any significant flaws or questionable points within the data presented and the method is described in sufficient detail to enable replication.

The 'activation' of decomposition by Mn oxidation at the oxic-anoxic interface allowing attack of litter by a wide spectrum of microbial pathways, rather than the specialist oxidative depolymerization enzymes, is an important finding which should drive a significant new parameterisation of soil biogeochemical models.

The specific experimental setup used here created a distinct 'hot moment' which the authors determine is limited by Mn(II) oxidation within the oxic zone that regulates supply of Mn to the oxic-anoxic interface. In the experiments this takes c. 30 days to develop to peak activity, a timescale which in dynamic soil environments may accommodate several cycles of wetting-drying and hence shorter-lived oxic-anoxic interfaces. While the 'hotspot' element of the findings remains an interesting and important insight, the implications of this 'hot moment' are less well explored in the discussion either as a limitation of the experimental design, a consideration for modelling this phenomenon, or as a potential key focus for future research.

Specific points:

Line 340 doesn't make good sense - presumably the design is modified from reactors used by ref 53, but as written this doesn't scan correctly. What was the modification from the original design and why? (lines 341-348)

> Response:
> We thank the reviewer for this comment. We believe that the confusion about Line 340 was due to an incomplete sentence that the reviewer kindly pointed out. The following changes were made from the original design: (i) the material and size of the reactors were changed to accommodate an increase in soil sampling during reactor deconstruction and (ii) pore water samplers were inserted to allow for periodic sampling and measurement of dissolved Mn.

Changes:
Lines 342 – 344: We have changed the wording to clarify that we adopted a modified design of the reactors from Jones et al. (2018)[1], specifically through the expansion of the original dimensions using a different plastic material (polypropylene) that would accommodate our need for a larger sampling volume.

Line 347: Emphasized that the inclusion of pore water samplers was an additional change.

Line 377-378 - sentence introducing and describing the artificial soil solution appears incomplete / concatenated / duplicated.

Response:
We thank the author for making this suggestion and have modified the sentence.

Changes:
Lines 383-384: Removed the duplicated sentence at the end of line 383 and fixed the follow up in line 384.

Reviewer #2 (Remarks to the Author):

The present manuscript addresses an interesting and relevant topic concerning the role of manganese (Mn) availability in regulating litter decomposition across oxic-anoxic interfaces in terrestrial ecosystems. The authors designed efficient soil reactors to systematically investigate the hotspots of litter decomposition, with a specific focus on the interplay between reactive Mn species and microbial enzyme activity. They reported that stimulating microbial production of reactive manganese(III) at the oxic–anoxic interfaces simultaneously doubled the expression levels of diverse litter-decomposing enzymes, which in turn significantly accelerated the rate of litter decomposition. This is a novel and valuable finding that can substantially improve our current understanding of the complex mechanisms underlying litter decomposition, a key process controlling carbon cycling in terrestrial environments. Generally speaking, the manuscript is well-prepared, with a logical structure and a well-designed experimental framework that effectively tests the research hypotheses. However, I have one major concern regarding the litter material used in the study, the exclusive use of broadleaf litter, without the inclusion of needle litter. Given that needle litter differs significantly from broadleaf litter in terms of chemical composition (e.g., lignin content) and decomposition dynamics, this exclusion may limit the generalizability of the study's findings. Below are some specific comments and suggestions.

Using "Mn" or "Manganese" would be more precise than "metals." The current title lacks specificity, as it does not explicitly highlight the central role of Mn, which is the core focus of the study.

Response:
We agree with the reviewer that mentioning manganese specifically would add specificity and focus to the title of the manuscript

Changes:
Line 1: We changed the Title to specifically refer to "Manganese" instead of "Metals"

The Abstract is overly concise and lacks key experimental data. I suggest supplementing it with specific quantitative results to better showcase the study's contributions and enhance the Abstract's informativeness.

> Response:
> We thank the reviewer for this suggestion and agree that adding quantitative information would showcase the novel findings relating reactive Mn to litter decomposition. We added quantitative information to the results statement while being mindful of the word limit for the Abstract.
>
> Changes:
> Lines 24-25: Added specific percent change for decomposition and emphasized the multi-fold increase in gene expression.

Lines 26–28, the claim that "the assumption that oxidative enzymatic decomposition is maximized under fully oxic conditions" is widely accepted does not align with existing literature. In my opinion, numerous studies have demonstrated that many enzymes can maintain or even enhance their activity in partially anoxic environments, such as under snow cover or frozen conditions. For instance, several studies have reported that freeze-thaw cycles can promote enzyme activity by breaking down litter structure and increasing substrate accessibility.

> Response:
> We agree with the reviewer that recent literature has indicated greater oxidative potential under dynamic redox conditions, either along oxic-anoxic interfaces or during oxic-anoxic transitions.
>
> Changes:
> Lines 27-28: We revised the statement in the Abstract to better frame our study's contribution to the shifting paradigm on oxidative enzymatic decomposition.

The novelty of the study lies primarily in the role of Mn availability at oxic–anoxic interfaces, rather than in the regulation of enzyme activity during litter decomposition itself. Therefore, I suggest that the authors directly introduce Mn and its role in the first paragraph of the Introduction, rather than focusing solely on oxidative decomposition, to better highlight the study's unique contribution.

> Response:
> We agree that Mn should be introduced earlier in the Introduction, particularly alongside the role of redox active metals at oxic-anoxic interfaces.
>
> Changes:
> Line 40: We now introduce Mn and its importance in regulating oxidative decomposition in the first paragraph.

Line 100, the diffusion reactors and other Methods used to identify decomposition hotspots should be described in earlier paragraphs.

Response:
We agree with the reviewer and now highlight these specific diffusion reactors and their capability of establishing stable oxygen gradients for detailed observations and analysis earlier in the Introduction.

Changes:
Lines 71-72: We discuss the usage of these diffusion reactors and similar reactors earlier in the Introduction

Lines 109–121 currently appear in an inappropriate section; this content should be included in the Methods section, as it describes experimental procedures. The Results section should focus on presenting key observations and findings, rather than methodological details.

Response:
We agree with the reviewer that there are several instances in the referred section that are more appropriate for the approach section of the Introduction and the Methods section.

Changes:
Lines 102-111: We moved relevant experimental information from the results section to the approach paragraph in the Introduction as well as the Methods. The approach section now introduces the experiment conceptually, while the Methods section provides the relevant details.

The subsection starting at Line 160 is excessively long and difficult to follow. I recommend streamlining this section to emphasize the most important observations, rather than detailing every result. For example, the last two paragraphs of this subsection could be moved to a separate subsection to improve readability.

Response:
We agree with the reviewer that the last two paragraphs in this specific subsection can be further separated into their own subsections.

Changes:
Line 156: We have divided this subsection into two shorter subsections that were renamed accordingly. The first one focuses on gene expression patterns and the second on linkages between gene expression to Mn(III) formation and decomposition.

The limitations associated with the litter type used (i.e., the exclusion of needle litter) should be thoroughly discussed in the Discussion section, including how this may affect the interpretation and generalizability of the results.

Response:
We thank the reviewer for this comment. The effect of Mn availability on litter decomposition is well established for both deciduous[2–4] and coniferous forest

ecosystems[4–7], although Mn-driven decomposition is generally expected to be a stronger factor in coniferous system due to higher litter Mn content[8].

Changes:
Lines 47-48: We make it clear that Mn is an important driver of decomposition in both deciduous and coniferous systems in the Introduction.
Lines 304-308: We added a statement on the litter choice to the Discussion.

Lines 351–353, the authors should clearly explain the rationale for selecting the specific litter types used in the study. Detailed information on litter quality and Mn concentrations should be provided.

Response:
We chose to conduct our experiment with broadleaf litter from a deciduous forest (Harvard Forest LTER) because we had previously found evidence that Mn(III) is directly linked to litter decomposition across a soil moisture gradient[9]. As part of this study, the litter had already been well characterized and provided a suitable ecosystem context for this mechanistic study.

Changes:
Line 108: We clarify that the litter we use is broadleaf litter.
Lines 356-359: We provide a justification for the use of litter type and refer to its characteristics in the Methods section.

Finally, the reactor design should be briefly described with a clear diagram. A visual illustration would help readers quickly understand the experimental setup.

Response:
We agree that having a simplified diagram of the reactor would help orient readers.

Changes:
Line 345: We reference the schematic of the reactors in the Methods section

Supplementary Materials: We have added Supplementary Figure 11, which is a simplified schematic of the diffusion reactors.

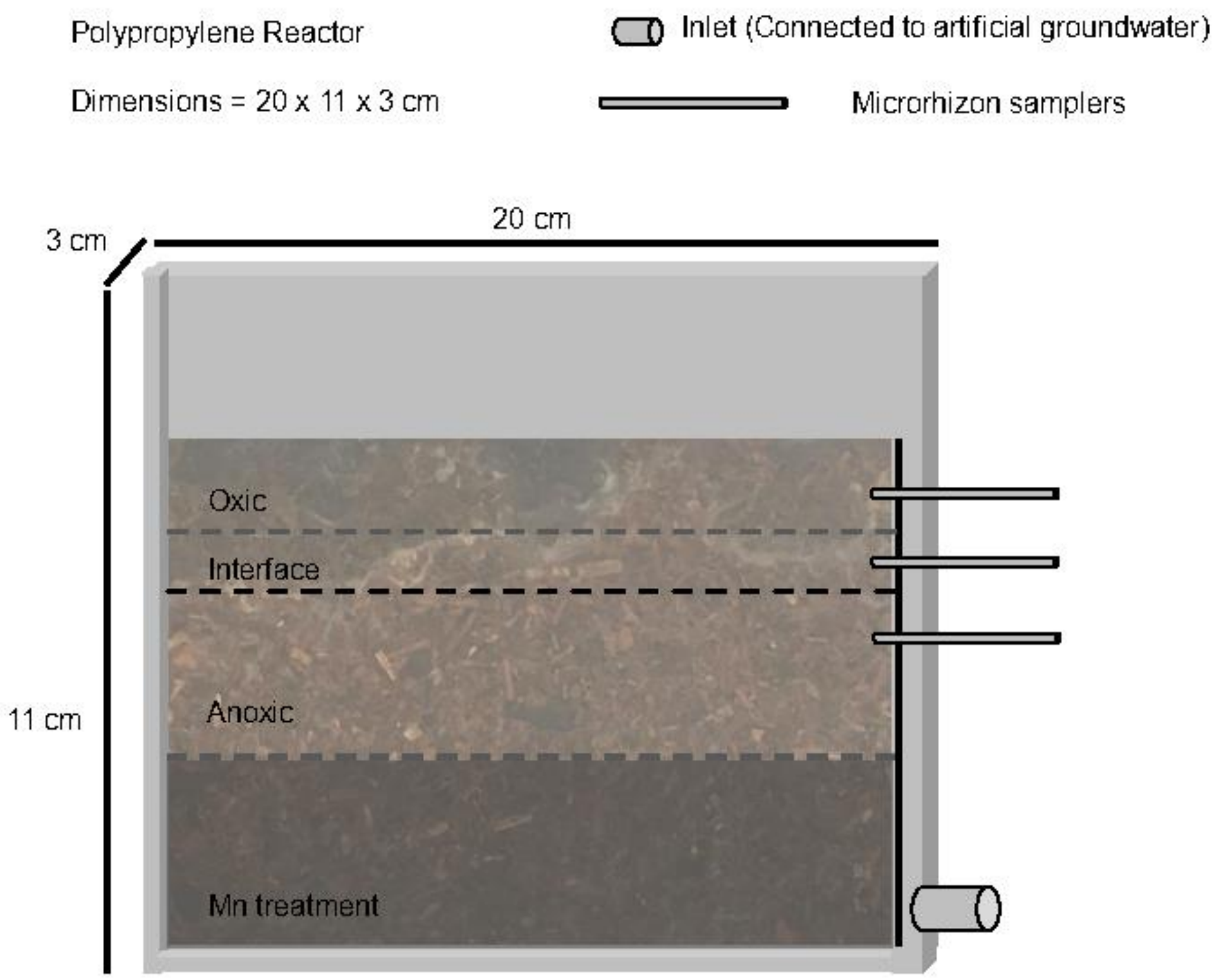

Supplementary Figure 11. Schematic of diffusion reactors Diffusion reactors were established using a polypropylene shell (20 x 11 x 3 cm). The total soil column height within the reactors was 75 mm. The oxic-anoxic interface was established by connecting the reactor to a source of artificial groundwater using an inlet at the bottom of the reactor and maintaining the water table height at 15 mm. We designated the top 0-10 mm below the soil surface as the oxic zone, 10-15 mm as the oxic-anoxic interface, and 15-50 mm as the anoxic zone. Microrhizon samplers were established at each sampling zone for porewater sampling. Depths 50-75 mm below the surface marked the bottom layer receiving Mn(III/IV) additions.

References:

1. Jones, M. E. *et al.* Manganese-Driven Carbon Oxidation at Oxic-Anoxic Interfaces. *Environ. Sci. Technol.* (2018) doi:10.1021/acs.est.8b03791.
2. Trum, F., Titeux, H., Ponette, Q. & Berg, B. Influence of manganese on decomposition of common beech (Fagus sylvatica L.) leaf litter during field incubation. *Biogeochemistry* (2015) doi:10.1007/s10533-015-0129-9.
3. Davey, M. P., Berg, B., Emmett, B. A. & Rowland, P. Decomposition of oak leaf litter is related to initial litter Mn concentrations. *Can. J. Bot.* **85**, 16–24 (2007).
4. Berg, B., Steffen, K. T. & McClaugherty, C. Litter decomposition rate is dependent on litter Mn concentrations. *Biogeochemistry* (2007) doi:10.1007/s10533-006-9050-6.
5. Berg, B. *et al.* Manganese in the litter fall-forest floor continuum of boreal and temperate pine and spruce forest ecosystems - A review. *Forest Ecology and Management* vol. 358 248–260 at https://doi.org/10.1016/j.foreco.2015.09.021 (2015).
6. Stendahl, J., Berg, B. & Lindahl, B. D. Manganese availability is negatively associated with carbon storage in northern coniferous forest humus layers. *Sci. Rep.* (2017) doi:10.1038/s41598-017-15801-y.

7. Zhang, Y. *et al.* Exchangeable manganese regulates carbon storage in the humus layer of the boreal forest. *Proc. Natl. Acad. Sci. U. S. A.* **121**, 2318382121 (2024).
8. Berg, B. *et al.* Manganese dynamics in decomposing needle and leaf litter — a synthesis. *Can. J. For. Res.* **43**, 1127–1136 (2013).
9. Jones, M. E. *et al.* Enzymes, Manganese, or Iron? Drivers of Oxidative Organic Matter Decomposition in Soils. *Environ. Sci. Technol.* **54**, 14114–14123 (2020).
